# Prediction of small‐for‐gestational age and fetal growth restriction at routine ultrasound examination at 35–37 weeks' gestation

**DOI:** 10.1002/uog.29223

**Published:** 2025-04-26

**Authors:** M. Lopian, S. Prasad, E. Segal, A. Dotan, C. O. Ulusoy, A. Khalil

**Affiliations:** ^1^ Fetal Medicine Unit, St George's University Hospitals NHS Foundation Trust University of London London UK; ^2^ Faculty of Medicine Tel Aviv University Tel Aviv Israel; ^3^ Sheba Medical Center, Tel Hashomer Tel Aviv Israel; ^4^ Ministry of Health, Etlik City Hospital, Perinatology Department Ankara Turkey; ^5^ Vascular Biology Research Centre Molecular and Clinical Sciences Research Institute, St George's University of London London UK; ^6^ Twin and Multiple Pregnancy Centre for Research and Clinical Excellence, St George's University Hospital, St George's University of London London UK; ^7^ Fetal Medicine Unit, Liverpool Women's Hospital Liverpool UK

**Keywords:** abdominal circumference, estimated fetal weight, fetal biometry, fetal Doppler, fetal growth restriction, perinatal mortality, perinatal outcome, predictive accuracy, small neonate, small‐for‐gestational age, third‐trimester ultrasound

## Abstract

**Objective:**

To evaluate the performance of sonographic fetal biometry and Doppler parameters assessed at routine third‐trimester ultrasound examination for predicting small‐for‐gestational age (SGA) and fetal growth restriction (FGR).

**Methods:**

This was a retrospective cohort study of low‐risk singleton pregnancies undergoing routine ultrasound examination between 35 + 0 and 37 + 6 weeks' gestation and delivered at St George's University Hospital, London, UK, between December 2019 and February 2024. The study outcomes were SGA (birth weight < 5^th^ centile) and FGR (birth weight < 3^rd^ centile or birth weight < 10^th^ centile with composite adverse perinatal outcome). Composite adverse perinatal outcome comprised intrauterine death, neonatal death or admission to the neonatal intensive care unit. Demographic characteristics, estimated fetal weight (EFW) and abdominal circumference centiles, as well as Doppler indices, including pulsatility indices (PI) of the umbilical artery (UA), middle cerebral artery (MCA) and uterine artery (UtA) were evaluated. The cerebroplacental ratio (CPR) was calculated, and all indices were converted to multiples of the median (MoM). Multivariable logistic regression analysis was performed to identify and adjust for confounders. The area under the receiver‐operating‐characteristics curve (AUC) was used to evaluate the model's performance for predicting small neonates.

**Results:**

A total of 14 161 pregnancies were included in the study. The prevalence of SGA and FGR neonates was 3.1% and 1.5%, respectively. Independent predictors of SGA and FGR, respectively, were: EFW centile (adjusted odds ratio (aOR) 0.91 (95% CI, 0.90–0.92); *P* < 0.001 and aOR 0.90 (95% CI, 0.89–0.91); *P* < 0.001); AC centile (aOR 0.91 (95% CI, 0.90–0.92); *P* < 0.001 and aOR 0.91 (95% CI, 0.90–0.92); *P* <0.001); UA‐PI MoM (aOR 4.60 (95% CI, 2.19–9.64); *P* < 0.001 and aOR 2.53 (95% CI, 1.05–6.10); *P* = 0.038); MCA‐PI MoM (aOR 0.37 (95% CI, 0.20–0.70); *P* = 0.002 and aOR 0.26 (95% CI, 0.12–0.59); *P* = 0.001); CPR MoM (aOR 0.23 (95% CI, 0.13–0.42); *P* < 0.001 and aOR 0.25 (95% CI, 0.12–0.53); *P* < 0.001); and UtA‐PI MoM (aOR 2.54 (95% CI, 1.68–3.83); *P* < 0.001 and aOR 2.16 (95% CI, 1.31–3.58); *P* = 0.003). The EFW centile alone was associated with an AUC of 0.917 (95% CI, 0.907–0.929) for the prediction of SGA and 0.925 (95% CI, 0.908–0.939) for the prediction of FGR. This was similar to AUCs of around 0.92 for the prediction of SGA and AUCs of around 0.93 for the prediction of FGR when the EFW centile was combined with any Doppler parameters.

**Conclusions:**

Sonographic fetal biometry evaluation in the late third trimester can predict delivery of a neonate affected by SGA or FGR, including those at risk for adverse perinatal outcomes. In an unselected population, fetal arterial Doppler parameters were independent predictors of SGA and FGR, but the addition of Doppler parameters to fetal biometry did not improve prediction of the incidence of small neonates. © 2025 The Author(s). *Ultrasound in Obstetrics & Gynecology* published by John Wiley & Sons Ltd on behalf of International Society of Ultrasound in Obstetrics and Gynecology.

## INTRODUCTION

Neonates that are small‐for‐gestational age (SGA) are at higher risk of adverse perinatal outcomes, as well as short‐ and long‐term health complications^1^, ^2^, ^3^, ^4^. Prenatal identification of fetuses at risk of SGA improves perinatal outcomes because targeted interventions can be implemented, including enhanced antepartum surveillance and timed delivery^5^. The ability to accurately identify fetuses at risk of SGA is of paramount importance. False‐positive diagnoses can lead to unwarranted surveillance and obstetric interventions, which not only increases maternal morbidity and anxiety but also contributes to inefficient allocation of healthcare resources^6^. Conversely, failure to detect fetuses genuinely at risk of SGA can result in increased perinatal morbidity and mortality^7^. Early studies on the predictive performance of sonographic fetal biometry assessment, performed in the third trimester to predict SGA, reported sensitivity varying between 29% and 53%^8^, ^9^, ^10^. Further studies attempting to enhance the predictive accuracy of the detection of SGA have shown that conducting ultrasound examinations at 36–37 weeks' gestation, as opposed to 32 weeks, significantly improves predictive capability, with reported sensitivities of up to 70%^11^, ^12^, ^13^. Other studies have investigated the role of combining various maternal demographic characteristics, Doppler parameters and biochemical markers with fetal biometry to improve diagnostic accuracy. While some studies showed that this approach improves the diagnostic performance for detecting SGA^14^, ^15^, others did not^16^, ^17^, ^18^. Furthermore, while combined screening performs relatively well at predicting which neonates will be SGA in the preterm period, its performance for predicting SGA at term is significantly worse^19^.

Identifying fetuses that experience growth restriction closer to term is particularly useful, as their risks of stillbirth, neonatal death and adverse neurodevelopmental outcomes significantly increase with advancing gestation beyond term^20^. Meanwhile, the cost associated with the intervention, namely, indicated delivery, is relatively small, owing to the relatively favorable prognosis of neonates born after 37 weeks^21^, ^22^.

The aim of this study was to evaluate the performance of sonographic fetal biometry and Doppler parameters assessed at routine late third‐trimester ultrasound, for predicting SGA and fetal growth restriction (FGR) in an unselected population.

## METHODS

This was a retrospective cohort study of routinely collected data from a single tertiary referral center. All singleton pregnancies that underwent routine third‐trimester ultrasound between 35 + 0 and 37 + 6 weeks' gestation and delivered at St George's University Hospital, London, UK, between December 2019 and February 2024 were included in the study. Cases were identified by searching the ultrasound electronic database (ViewPoint version 5.6.26.148; ViewPoint Bildverarbeitung GmbH, Weßling, Germany) at the Fetal Medicine Unit of St George's Hospital. Pregnancies complicated by major fetal abnormalities, aneuploidy or genetic syndrome, and those with missing pregnancy outcome data were excluded from the analysis. Gestational age (GA) was calculated using crown–rump length measurement^23^ taken at 11–13 weeks for pregnancies conceived spontaneously and from the date of embryo transfer for those conceived via assisted reproductive technology (ART). The following maternal demographic characteristics were extracted from electronic hospital records: maternal age; ethnicity; nulliparity; previous Cesarean delivery; previous stillbirth; maternal height, weight and body mass index; smoking or alcohol consumption at booking; use of ART for conception; and development of gestational diabetes or hypertensive disorders of pregnancy (HDP).

In 2019, St George's Hospital adopted a policy of universal routine third‐trimester ultrasound examination at 36 weeks, which involves: fetal biometry measurements, including fetal head circumference, abdominal circumference (AC) and femur length; assessment of fetal Doppler measurements, including those of the umbilical arteries (UA), middle cerebral artery (MCA) and uterine arteries (UtA); and examination of fetal presentation, amniotic fluid volume and placental location.

Routine fetal biometry was measured in line with the International Society of Ultrasound in Obstetrics and Gynecology (ISUOG) guidelines^24^, and estimated fetal weight (EFW) was calculated using the Hadlock‐3 formula^25^. AC and EFW were converted into centiles according to GA^26^, ^27^. Oligohydramnios was diagnosed when the deepest vertical pocket of amniotic fluid measured was ≤ 2 cm^24^. Polyhydramnios was diagnosed when the amniotic fluid index was ≥ 25 cm^24^. UA, MCA and UtA Doppler waveforms were recorded using color Doppler and pulsed Doppler as per ISUOG standards^24^, and the pulsatility index (PI) was calculated. The UA was examined in a free loop of the umbilical cord and the MCA was examined at its proximal one‐third where it originates from the internal carotid artery, with the angle of insonation as close as possible to zero, at the point at which it passes the sphenoid wing, close to the circle of Willis. UtA Doppler was recorded by placing the transducer over the iliac fossa and identifying the UtA as it crosses the external iliac artery, 1 cm downstream from the crossover point^24^. Pulsed Doppler was then applied and PI was measured using three similar waveforms. The cerebroplacental ratio (CPR) was calculated as the ratio between MCA‐PI and UA‐PI. The mean value of the left and right UtA‐PI was calculated. All Doppler indices were converted into multiples of the median (MoM)^28^, ^29^, ^30^. If a woman had more than one sonographic fetal biometry evaluation after 35 weeks, only the first examination was included in the analysis to ensure that the scan was performed for a routine indication. Birth‐weight values were converted into centiles according to neonatal sex and GA at delivery^31^. According to national guidelines, birth was recommended by 39 + 6 weeks when the EFW was between the 3^rd^ and 10^th^ centiles and by 37 + 6 weeks when the EFW was below the 3^rd^ centile^32^.

### Outcome measures

The primary outcome was prediction of SGA, defined as birth weight < 5^th^ centile. A secondary outcome was prediction of FGR, defined as birth weight < 3^rd^ centile or birth weight < 10^th^ centile with a composite adverse perinatal outcome. A composite adverse perinatal outcome comprised intrauterine death (fetal death prior to delivery), neonatal death (death within the first 28 days after delivery) or admission to the neonatal intensive care unit (NICU).

### Statistical analysis

Continuous variables are described using median and interquartile range (IQR), while categorical and binary variables are given as *n* (%). The Shapiro–Wilk test was applied to examine the normality of the data. Differences between groups were analyzed using the independent samples *t*‐test, Mann–Whitney *U*‐test or the chi‐square/Fisher's exact test, as appropriate. First, we explored the difference in ultrasound measurements between pregnancies with and those without SGA and FGR, separately. Subsequently, univariable and multivariable (using the enter method) binary logistic regression analysis was performed to identify potential independent predictors for the outcome of interest. Results are reported as adjusted odds ratios (aOR). Odds ratio estimates in the regression analyses were reported for one standard unit change in each variable. Procedures such as checking for multicollinearity were included in standard postestimation diagnostics. Adjustments were made for confounding factors including maternal age, parity, smoking status, presence of HDP, ethnicity and GA at delivery. The predictive performance of individual variables, as well as various combinations of variables, for the outcomes SGA and FGR were evaluated. Model fit was assessed using the Hosmer–Lemeshow test. Predictive accuracy measures (sensitivity, positive and negative predictive value) were calculated for a 10% false‐positive rate to facilitate comparisons between models. Calibration plots were constructed for each model to compare the observed *vs* the expected outcome rates, providing a visual assessment of calibration. Internal validation of the area under the receiver‐operating‐characteristics curve (AUC) for each predictive model was performed using bootstrapping, generating 95% CIs based on the 2.5^th^ and 97.5^th^ centiles of the bootstrap distribution. Overall model performance was evaluated using the Hosmer–Lemeshow test, C‐statistic, calibration plots and bootstrap‐based CI for all key metrics. Analysis was carried out using SPSS version 24 (SPSS Inc., Chicago, IL, USA). *P* < 0.05 was taken to indicate statistical significance.

## RESULTS

A total of 18 349 singleton pregnancies underwent routine third‐trimester ultrasound during the study period. After excluding patients with missing outcome data, fetal anomalies and repeat ultrasound scans, 14 161 pregnancies were included in the final analysis. There were 12 (0.1%) intrauterine deaths, 3 (0.02%) neonatal deaths and 278 (2.0%) neonates admitted to the NICU. Median maternal age was 35 (IQR, 31–38) years, while the median GA at ultrasound was 36.6 (IQR, 36.2–36.7) weeks and at delivery it was 39.9 (IQR, 39.0–40.6) weeks. The median interval between ultrasound scan and delivery was 23 (17–29) days and the median birth‐weight centile was 46.7 (IQR, 25.3–70.5). There were 433 (3.1%) neonates with SGA and 215 (1.5%) neonates with FGR. Tables 1 and 2 present the demographic, fetal biometry and Doppler characteristics of the patients with *vs* those without SGA and FGR, respectively.

**Table 1:** Demographic, fetal biometry and Doppler characteristics of 14 161 singleton pregnancies that underwent routine third‐trimester ultrasound at 35–37 weeks, according to whether they had a small‐for‐gestational‐age (SGA) neonate (birth weight < 5^th^ centile)

Characteristic	SGA (*n* = 433)	Not SGA (*n* = 13 728)*	*P*
Maternal age (years)	34.7 (29.2–37.7)	35.3 (31.8–38.3)	< 0.001
Maternal BMI at booking (kg/m^2^)	24.0 (21.0–28.0)	24.0 (22.0–28.0)	< 0.001
Nulliparous	291 (67.2)	6913 (50.4)	< 0.001
Maternal ethnicity			
White	144 (33.3)	6748 (49.2)	< 0.001
Black	50 (11.5)	1469 (10.7)	0.287
Asian	138 (31.9)	2212 (16.1)	< 0.001
Other	101 (23.3)	3299 (24.0)	0.368
ART	22 (5.1)	689 (5.0)	0.476
Previous CS	6 (1.4)	152 (1.1)	0.293
Previous stillbirth	0 (0)	28 (0.2)	N/A
Smoking during pregnancy	34 (7.9)	409 (3.0)	< 0.001
Alcohol consumption during pregnancy	1 (0.2)	69 (0.5)	0.219
GDM	42 (9.7)	1561 (11.4)	0.140
HDP	26 (6.0)	319 (2.3)	< 0.001
Birth weight (g)	2440 (2255–2580)	3400 (3120–3700)	< 0.001
Birth‐weight centile	3.3 (2.0–4.2)	47.9 (27.2–71.0)	< 0.001
Induction of labor	249 (57.5)	4769 (34.7)	< 0.001
GA at scan (weeks)	36.4 (36.2–36.7)	36.6 (36.3–36.7)	0.212
GA at birth (weeks)	39.0 (37.7–39.9)	39.9 (39.1–40.6)	< 0.001
Scan‐to‐birth interval (weeks)	2.6 (1.3–3.3)	3.3 (2.6–4.1)	< 0.001
UA‐PI MoM	1.03 (0.91–1.12)	0.94 (0.84–1.03)	< 0.001
MCA‐PI MoM	1.12 (1.06–1.33)	1.25 (1.13–1.38)	< 0.001
CPR MoM	0.95 (0.82–1.10)	1.06 (0.94–1.20)	< 0.001
UtA‐PI MoM	1.00 (0.82–1.25)	0.92 (0.78–1.10)	< 0.001
EFW centile	20.60 (10.78–34.77)	62.22 (46.06–77.4)	< 0.001
AC centile	19.00 (9.40–31.10)	56.20 (39.0–72.90)	< 0.001
Oligohydramnios	3 (0.7)	20 (0.1)	0.032
Polyhydramnios	0 (0)	85 (0.6)	0.117

Data are given as median (interquartile range) or *n* (%).

* Non‐SGA was defined as birth weight ≥ 5^th^ centile and included some cases defined as growth restricted (those with birth weight ≥ 5^th^ centile and < 10^th^ centile with adverse outcome).

AC, abdominal circumference; ART, assisted reproductive technology; BMI, body mass index; CPR, cerebroplacental ratio; CS, Cesarean section; EFW, estimated fetal weight; GA, gestational age; GDM, gestational diabetes mellitus; HDP, hypertensive disorders of pregnancy; MCA, middle cerebral artery; MoM, multiples of the median; N/A, not applicable; PI, pulsatility index; UA, umbilical artery; UtA, uterine artery.

**Table 2:** Demographic, fetal biometry and Doppler characteristics of 14 161 singleton pregnancies that underwent routine third‐trimester ultrasound at 35–37 weeks, according to whether they had a growth‐restricted neonate

Characteristic	FGR* (*n* = 215)	Not FGR (*n* = 13 946)†	*P*
Maternal age (years)	33.0 (29.2–37.7)	35.2 (31.8–38.7)	< 0.001
Maternal BMI at booking (kg/m^2^)	24.0 (21.0–28.0)	24.0 (22.0–28.0)	0.093
Nulliparous	142 (66.0)	7062 (50.6)	< 0.001
Maternal ethnicity			
White	72 (33.5)	6820 (48.9)	< 0.001
Black	21 (9.8)	1498 (10.7)	0.323
Asian	72 (33.5)	2278 (16.3)	< 0.001
Other	50 (23.3)	3350 (24.0)	0.397
ART	11 (5.1)	700 (5.0)	0.951
Previous CS	5 (2.3)	153 (1.1)	0.094
Previous stillbirth	0 (0)	28 (0.2)	N/A
Smoking during pregnancy	22 (10.2)	421 (3.0)	< 0.001
Alcohol consumption during pregnancy	1 (0.5)	69 (0.5)	0.471
GDM	16 (7.4)	1587 (11.4)	0.021
HDP	14 (6.5)	331 (2.4)	< 0.001
Birth weight (g)	2340 (2170–2480)	3400 (3100–3700)	< 0.001
Birth‐weight centile	2.0 (1.30–2.70)	47.40 (26.30–70.70)	< 0.001
Induction of labor	122 (56.7)	4896 (35.1)	< 0.001
GA at scan (weeks)	36.4 (36.1–36.6)	36.6 (36.3–36.7)	0.091
GA at birth (weeks)	38.9 (37.6–39.9)	39.9 (39.0–40.6)	< 0.001
Scan‐to‐birth interval (weeks)	2.3 (1.1–3.3)	3.3 (2.6–4.1)	< 0.001
UA‐PI MoM	0.99 (0.90–1.12)	0.94 (0.84–1.04)	< 0.001
MCA‐PI MoM	1.18 (1.04–1.30)	1.24 (1.13–1.38)	< 0.001
CPR MoM	0.93 (0.84–1.09)	1.08 (0.94–1.20)	< 0.001
UtA‐PI MoM	1.01 (0.82–1.28)	0.91 (0.78–1.08)	0.002
EFW centile	16.98 (8.21–32.10)	61.80 (45.29–77.16)	< 0.001
AC centile	15.30 (6.20–28.03)	56.00 (38.70–32.50)	< 0.001
Oligohydramnios	1 (0.5)	22 (0.2)	0.299
Polyhydramnios	0 (0)	85 (0.6)	0.643

Data are given as median (interquartile range) or *n* (%).

* Fetal growth restriction (FGR) defined as birth weight < 3^rd^ centile or < 10^th^ centile with composite adverse perinatal outcome.

† Non‐FGR defined as neonate with birth weight ≥ 10^th^ centile or 3^rd^–10^th^ centile without adverse outcome.

AC, abdominal circumference; ART, assisted reproductive technology; BMI, body mass index; CPR, cerebroplacental ratio; CS, Cesarean section; EFW, estimated fetal weight; GA, gestational age; GDM, gestational diabetes mellitus; HDP, hypertensive disorders of pregnancy; MCA, middle cerebral artery; MoM, multiples of the median; N/A, not applicable; PI, pulsatility index; UA, umbilical artery; UtA, uterine artery.

### Prediction of SGA

**Figure 1:**
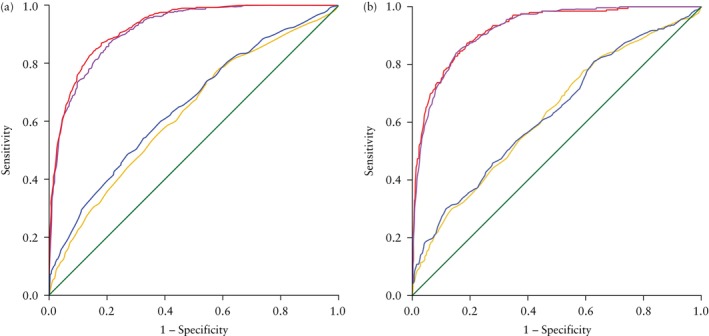
Receiver‐operating‐characteristics curves for prediction of small‐for‐gestational‐age neonate (birth weight < 5^th^ centile) (a) and growth‐restricted neonate (birth weight < 3^rd^ centile or < 10^th^ centile with composite adverse perinatal outcome) (b) by maternal characteristics (maternal age, nulliparity, smoking and hypertensive disorders of pregnancy) (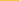), Doppler umbilical artery pulsatility index (UA‐PI) (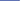), estimated fetal weight (EFW) centile (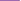) and combination of UA‐PI and EFW centile (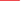).

Women who delivered a neonate that was SGA (birth weight < 5^th^ centile) were more likely to be nulliparous (67.2% *vs* 50.4%; *P* < 0.001) and of Asian ethnicity (31.9% *vs* 16.1%; *P* < 0.001), to have smoked during pregnancy (7.9% *vs* 3.0%; *P* < 0.001) and to have HDP (6.0% *vs* 2.3%; *P* < 0.001). They were also more likely to have undergone induction of labor (57.5% *vs* 34.7%; *P* < 0.001), and delivered 6 days earlier than neonates that were not SGA (39 + 0 *vs* 39 + 6; *P* < 0.001). Median AC centile (19.00 *vs* 56.20; *P* < 0.001), EFW centile (20.60 *vs* 62.22; *P* < 0.001), MCA‐PI MoM (1.12 *vs* 1.25; *P* < 0.001) and CPR MoM (0.95 *vs* 1.06; *P* < 0.001) were significantly lower in SGA fetuses compared with the non‐SGA group, whereas UA‐PI MoM (1.03 *vs* 0.94; *P* < 0.001) and UtA‐PI MoM (1.00 *vs* 0.92; *P* < 0.001) were significantly higher in these cases compared with the non‐SGA group (Table 1). The results of the univariable and multivariable analyses with aOR for each variable for the prediction of SGA < 5^th^ centile are presented in Table 3. The following sonographic parameters were found to be significant predictors of SGA after adjusting for confounders: EFW centile (aOR 0.91 (95% CI, 0.90–0.92); *P* < 0.001), AC centile (aOR 0.91 (95% CI, 0.90–0.92); *P* < 0.001), UA‐PI MoM (aOR 4.60 (95% CI, 2.19–9.64); *P* < 0.001), MCA‐PI MoM (aOR 0.37 (95% CI, 0.20–0.70); *P* = 0.002), CPR MoM (aOR 0.23 (95% CI, 0.13–0.42); *P* < 0.001) and UtA‐PI MoM (aOR 2.54 (95% CI, 1.68–3.83); *P* < 0.001). Multiple models were constructed for the prediction of SGA < 5^th^ centile. The predictive performance and accuracy of these models are presented in Table 4. EFW and AC centiles were both independently able to predict SGA with a high degree of accuracy (AUC of 0.917 (95% CI, 0.907–0.929) and 0.893 (95% CI, 0.879–0.906), respectively). Maternal characteristics (combination of maternal age, nulliparity, smoking and HDP) and individual Doppler parameters had a poorer predictive performance (AUC ranging from 0.587 to 0.663) (Table 4, Figure 1a). The combination of EFW centile and various Doppler parameters did not significantly improve the predictive accuracy for SGA (AUC ranging from 0.919 to 0.921) compared with the EFW centile or AC centile alone (Table 4, Figure 1a). Calibration plots for each of the models are shown in Figures S1–S13.

**Table 3:** Univariable and multivariable logistic regression analyses for association of maternal, pregnancy and fetal characteristics with delivery of small‐for‐gestational‐age (SGA) neonate (birth weight < 5^th^ centile)

Variable	OR (95% CI)	*P*	aOR (95% CI)*	*P*
Maternal age (years)	0.96 (0.94–0.97)	< 0.001	1.02 (0.997–1.04)	0.083
Ethnicity				
White	0.71 (0.54–0.93)	0.012	0.80 (0.58–1.12)	0.197
Black	1.13 (0.79–1.61)	0.514	1.18 (0.77–1.82)	0.441
Asian	2.06 (1.56–2.72)	< 0.001	1.46 (1.04–2.07)	0.031
Other	1.15 (0.68–1.92)	0.609	0.99 (0.52–1.89)	0.984
Nulliparous	2.01 (1.64–2.46)	< 0.001	2.16 (1.75–2.67)	< 0.001
Previous CS	0.80 (0.35–1.82)	0.592		
ART	0.99 (0.64–1.53)	0.963		
Smoking	2.77 (1.92–3.98)	< 0.001	3.81 (2.62–5.63)	< 0.001
BMI (kg/m^2^)	0.96 (0.94–0.98)	< 0.001	0.99 (0.97–1.02)	0.468
GDM	1.17 (0.85–1.60)	0.346		
HDP	2.64 (1.75–4.00)	< 0.001	2.27 (1.27–4.04)	0.006
GA at scan (weeks)	0.81 (0.61–1.09)	0.162		
AC centile	0.91 (0.90–0.92)	< 0.001	0.91 (0.90–0.92)	< 0.001
EFW centile	0.90 (0.90–0.91)	< 0.001	0.91 (0.90–0.92)	< 0.001
UA‐PI MoM	74.56 (39.53–140.63)	< 0.001	4.60 (2.19–9.64)	< 0.001
MCA‐PI MoM	0.16 (0.09–0.28)	< 0.001	0.37 (0.20–0.70)	0.002
CPR MoM	0.02 (0.01–0.04)	< 0.001	0.23 (0.13–0.42)	< 0.001
UtA‐PI MoM	5.94 (4.30–8.21)	< 0.001	2.54 (1.68–3.83)	< 0.001
Oligohydramnios	0.21 (0.06–0.71)	0.012	0.59 (0.13–2.16)	0.485
GA at birth (weeks)	0.52 (0.48–0.56)	< 0.001	1.01 (0.91–1.12)	0.851

* Adjusted for maternal age, nulliparity, smoking, ethnicity, hypertensive disorders of pregnancy (HDP), body mass index (BMI) and gestational age (GA) at birth.

High multicollinearity was observed between umbilical artery (UA) pulsatility index (PI) multiples of the median (MoM), middle cerebral artery (MCA)‐PI MoM and cerebroplacental ratio (CPR) MoM; therefore, adjusted odds ratios (aORs) were calculated in separate models to estimate their independent effects.

A similar approach was applied for calculating aORs for abdominal circumference (AC) and estimated fetal weight (EFW) centiles, which also showed high multicollinearity.

aORs derived from model with best fit and discriminative capacity.

ART, assisted reproductive technology; CS, Cesarean section; GDM, gestational diabetes mellitus; OR, odds ratio; UtA, uterine artery.

**Table 4:** Performance of prediction models for delivery of small‐for‐gestational‐age (SGA) neonate (birth weight < 5^th^ centile)

Model	AUC (95% CI)	*P*	Hosmer– Lemeshow *P*	Sensitivity (%)	PPV (%)	NPV (%)
Maternal characteristics*	0.631 (0.603–0.657)	< 0.001	0.004	21.7	6.4	96.3
EFW centile	0.917 (0.907–0.929)	< 0.001	0.547	73.7	18.9	99.1
AC centile	0.893 (0.879–0.906)	< 0.001	0.134	66.1	17.3	98.8
UA‐PI	0.645 (0.617–0.672)	< 0.001	0.231	25.9	7.6	97.5
MCA‐PI	0.587 (0.556–0.616)	< 0.001	0.065	18.7	5.9	97.2
CPR	0.663 (0.635–0.689)	< 0.001	0.009	28.0	8.1	97.6
UtA‐PI	0.604 (0.575–0.635)	< 0.001	< 0.001	24.9	7.6	97.4
EFW centile + UA‐PI MoM	0.919 (0.907–0.930)	< 0.001	0.251	73.7	18.7	99.1
EFW centile + CPR MoM	0.921 (0.910–0.932)	< 0.001	0.488	73.2	18.8	99.1
EFW centile + UA‐PI MoM + MCA‐PI MoM	0.921 (0.911–0.933)	< 0.001	0.397	73.0	18.9	99.1
EFW centile + UA‐PI MoM + UtA‐PI MoM	0.919 (0.908–0.930)	< 0.001	0.042	73.4	19.2	99.1
EFW centile + CPR + UtA‐PI MoM	0.921 (0.910–0.931)	< 0.001	0.629	73.0	19.4	99.1
EFW centile + MCA‐PI MoM + UtA‐PI MoM	0.921 (0.911–0.933)	< 0.001	0.404	73.2	19.2	99.1

* Maternal age, nulliparity, smoking and hypertensive disorders of pregnancy.

Hosmer–Lemeshow *P* > 0.05 implies model goodness‐of‐fit.

Sensitivity, positive predictive value (PPV) and negative predictive value (NPV) were calculated for a 10% false‐positive rate.

AC, abdominal circumference; AUC, area under receiver‐operating‐characteristics curve; CPR, cerebroplacental ratio; EFW, estimated fetal weight; MCA, middle cerebral artery; MoM, multiples of the median; PI, pulsatility index; UA, umbilical artery; UtA, uterine artery.

### Prediction of FGR

Women who delivered a neonate affected by FGR (birth weight < 3^rd^ centile or < 10^th^ centile with composite adverse perinatal outcome) were more likely to be nulliparous (66.0% *vs* 50.6%; *P* < 0.001) and of Asian ethnicity (33.5% *vs* 16.3%; *P* < 0.001), to have smoked during pregnancy (10.2% *vs* 3.0%; *P* < 0.001) and to have HDP (6.5% *vs* 2.4%; *P* < 0.001). These patients also had a significantly higher rate of induction of labor (56.7% *vs* 35.1%; *P* < 0.001) and were delivered 1 week earlier than the non‐FGR group (38 + 6 *vs* 39 + 6 weeks; *P* < 0.001). Median AC centile (15.30 *vs* 56.00; *P* < 0.001), EFW centile (16.98 *vs* 61.80; *P* < 0.001), MCA‐PI MoM (1.18 *vs* 1.24; *P* < 0.001) and CPR MoM (0.93 *vs* 1.08; *P* < 0.001) were significantly lower in those with FGR, whereas UA‐PI MoM (0.99 *vs* 0.94; *P* < 0.001) and UtA‐PI MoM (1.01 *vs* 0.91; *P* = 0.002) were significantly higher in those with FGR compared with the non‐FGR group (Table 2). The results of univariable and multivariable analysis with aOR for each variable for the prediction of FGR are presented in Table 5. The following sonographic parameters were found to be significant predictors of FGR after adjusting for confounders: AC centile (aOR 0.91 (95% CI, 0.90–0.92); *P* < 0.001), EFW centile (aOR 0.90 (95% CI, 0.89–0.91); *P* < 0.001), UA‐PI MoM (aOR 2.53 (95% CI, 1.05–6.10); *P* = 0.038), MCA‐PI MoM (aOR 0.26 (95% CI, 0.12–0.59); *P* = 0.001), CPR MoM (aOR 0.25 (95% CI, 0.12–0.53); *P* < 0.001) and UtA‐PI MoM (aOR 2.16 (95% CI, 1.31–3.58); *P* = 0.003). Multiple models were constructed for the prediction of FGR. The predictive performance and accuracy of these models are presented in Table 6. EFW and AC centiles were both independently able to predict FGR with a high degree of accuracy (AUC of 0.925 (95% CI, 0.908–0.939) and 0.906 (95% CI, 0.886–0.925), respectively). Maternal characteristics (combination of maternal age, nulliparity, smoking and HDP) and individual Doppler parameters had a poorer predictive performance (AUC ranging from 0.598 to 0.671). The combination of EFW centile and various Doppler parameters did not significantly improve the predictive accuracy (AUC ranging from 0.925 to 0.928) compared with the EFW centile or AC centile alone (Table 6, Figure 1b). Calibration plots for each of the models are presented in Figures S14–S26.

**Table 5:** Univariable and multivariable logistic regression analyses for association of maternal, pregnancy and fetal characteristics with delivery of growth‐restricted neonate*

Variable	OR (95% CI)	*P*	aOR (95% CI)†	*P*
Maternal age (years)	0.96 (0.93–0.98)	0.001	1.03 (0.99–1.06)	0.101
Ethnicity				
White	0.80 (0.54–1.18)	0.256	0.86 (0.56–1.34)	0.512
Black	1.04 (0.61–1.78)	0.881	0.93 (0.52–1.67)	0.805
Asian	2.38 (1.60–3.54)	< 0.001	1.57 (1.00–2.45)	0.049
Other	1.70 (0.88–3.27)	0.115	1.41 (0.64–3.09)	0.391
Nulliparous	1.89 (1.42–2.50)	< 0.001	2.14 (1.52–2.83)	< 0.001
Previous CS	0.47 (0.19–1.16)	0.101		
ART	0.99 (0.54–1.82)	0.974		
Smoking	3.62 (2.31–5.69)	< 0.001	5.00 (3.13–8.12)	< 0.001
BMI (kg/m^2^)	0.98 (0.95–1.00)	0.085		
GDM	1.61 (0.98–2.69)	0.067		
HDP	2.80 (1.61–4.87)	< 0.001	2.20 (1.08–4.49)	0.030
GA at scan (weeks)	0.73 (0.49–1.11)	0.140		
AC centile	0.90 (0.89–0.91)	< 0.001	0.91 (0.90–0.92)	< 0.001
EFW centile	0.90 (0.89–0.91)	< 0.001	0.90 (0.89–0.91)	< 0.001
UA‐PI MoM	59.83 (25.88–138.24)	< 0.001	2.53 (1.05–6.10)	0.038
MCA‐PI MoM	0.08 (0.04–0.18)	< 0.001	0.26 (0.12–0.59)	0.001
CPR MoM	0.02 (0.01–0.04)	< 0.001	0.25 (0.12–0.53)	< 0.001
UtA‐PI MoM	6.15 (3.99–9.47)	< 0.001	2.16 (1.31–3.58)	0.003
Oligohydramnios	0.34 (0.05–2.54)	0.294		
GA at birth (weeks)	0.50 (0.45–0.56)	< 0.001	1.14 (0.98–1.31)	0.081

* Fetal growth restriction defined as birth weight < 3^rd^ centile or < 10^th^ centile with composite adverse perinatal outcome.

† Adjusted for maternal age, nulliparity, smoking, ethnicity, hypertensive disorders of pregnancy (HDP), body mass index (BMI) and gestational age (GA) at birth.

High multicollinearity was observed between umbilical artery (UA) pulsatility index (PI) multiples of the median (MoM), middle cerebral artery (MCA)‐PI MoM and cerebroplacental ratio (CPR) MoM; therefore, adjusted odds ratios (aORs) were calculated in separate models to estimate their independent effects.

A similar approach was applied for calculating aORs for abdominal circumference (AC) and estimated fetal weight (EFW) centiles, which also showed high multicollinearity.

aORs derived from model with best fit and discriminative capacity.

ART, assisted reproductive technology; CS, Cesarean section; GDM, gestational diabetes mellitus; OR, odds ratio; UtA, uterine artery.

**Table 6:** Performance of prediction models for delivery of growth‐restricted neonate*

Model	AUC (95% CI)	*P*	Hosmer– Lemeshow *P*	Sensitivity (%)	PPV (%)	NPV (%)
Maternal characteristics†	0.630 (0.595–0.666)	< 0.001	0.305	24.2	3.6	98.7
EFW centile	0.925 (0.908–0.939)	< 0.001	0.08	74.9	10.4	99.6
AC centile	0.906 (0.886–0.925)	< 0.001	0.154	72.1	10.1	99.5
UA‐PI	0.623 (0.583–0.664)	< 0.001	0.046	23.7	4.0	98.7
MCA‐PI	0.611 (0.565–0.650)	< 0.001	0.101	21.9	3.5	98.7
CPR	0.671 (0.634–0.708)	< 0.001	0.115	25.0	3.7	98.7
UtA‐PI	0.598 (0.553–0.638)	< 0.001	< 0.001	27.0	4.1	98.8
EFW centile + UA‐PI MoM	0.925 (0.908–0.942)	< 0.001	0.169	75.8	10.6	99.6
EFW centile + CPR MoM	0.927 (0.910–0.942)	< 0.001	0.339	77.4	10.6	99.6
EFW centile + UA‐PI MoM + MCA‐PI MoM	0.927 (0.910–0.942)	< 0.001	0.322	77.2	10.8	99.6
EFW centile + UA‐PI MoM + UtA‐PI MoM	0.925 (0.910–0.941)	< 0.001	0.311	76.3	10.5	99.6
EFW centile + CPR + UtA‐PI MoM	0.927 (0.911–0.944)	< 0.001	0.317	76.9	10.5	99.6
EFW centile + MCA‐PI MoM + UtA‐PI MoM	0.928 (0.912–0.944)	< 0.001	0.452	77.2	10.8	99.6

* Fetal growth restriction defined as birth weight < 3^rd^ centile or < 10^th^ centile with composite adverse perinatal outcome.

† Maternal age, nulliparity, smoking and hypertensive disorders of pregnancy.

Hosmer–Lemeshow *P* > 0.05 implies model goodness‐of‐fit.

Sensitivity, positive predictive value (PPV) and negative predictive value (NPV) were calculated for a 10% false‐positive rate.

AC, abdominal circumference; AUC, area under receiver‐operating‐characteristics curve; CPR, cerebroplacental ratio; EFW, estimated fetal weight; MCA, middle cerebral artery; MoM, multiples of the median; PI, pulsatility index; UA, umbilical artery; UtA, uterine artery.

## DISCUSSION

### Summary of key findings

The results of this study demonstrate that fetal biometry evaluated during a late third‐trimester ultrasound examination shows excellent predictive capabilities for detecting SGA and FGR. The predictive performance of fetal biometry was superior to that of Doppler parameters or maternal characteristics alone, and predictive models combining Doppler and fetal biometry parameters did not significantly improve the predictive accuracy compared with fetal biometry alone.

### Interpretation of study findings and comparison with published literature

Studies investigating the prediction of SGA based on the sonographic assessment of fetal biometry alone in the third trimester have reported detection rates (DR) of 50–70%^8^, ^9^, ^10^. To further enhance predictive accuracy, studies have explored whether combined screening models, incorporating combinations of demographic, clinical and sonographic parameters, improve prediction of the detection of SGA neonates. A prospective study of 1590 singleton pregnancies evaluated between 32 + 0 and 36 + 6 weeks demonstrated that the combination of maternal demographic characteristics, EFW centile, UtA‐PI and serum markers offered superior predictive accuracy of SGA at term compared with the EFW centile alone (DR, 61% *vs* 52%)^14^. However, the overall predictive performance was still considered modest, even with the combined approach.

In contrast, another study evaluating 19 209 low‐risk pregnancies at 36 + 0 to 36 + 6 weeks demonstrated that maternal characteristics, EFW *Z*‐score, UtA‐PI MoM MCA‐PI MoM and placental growth factor (PlGF) MoM contributed independently to the prediction of SGA (birth weight < 10^th^ centile). However, adding Doppler parameters and PlGF to the existing predictive model based on maternal characteristics and EFW *Z*‐score alone did not improve predictive accuracy^15^. These results are in line with those of the current study and others that have failed to demonstrate added value for biomarkers of placental dysfunction such as UtA‐PI^17^, soluble fms‐like tyrosine kinase‐1^18^, PlGF^18^ or CPR^16^ for improving the sonographic prediction of SGA at term.

Our results may differ from those of Miranda *et al*.^14^ owing to the different GAs at ultrasound examination in the two studies. The median GA in the study of Miranda *et al*.^14^ was 33 + 0 weeks, whereas that in ours was 36 + 4 weeks, resulting in a longer scan‐to‐delivery interval.

There is considerable evidence that measuring EFW at 35–37 weeks is more accurate for detecting SGA than it is at 30–32 weeks^11^, ^12^, ^13^. Furthermore, preterm SGA is more accurately predicted than term SGA using combined third‐trimester screening models with Doppler parameters^15^, ^16^, ^17^, ^18^, ^19^.

It is plausible that, when the interval between ultrasound assessment and birth is short, as in our study (23 days), the predictive accuracy of biometry is high, thereby masking the added value of biomarkers of placental dysfunction. On the other hand, there may be a potential role for Doppler parameters to bridge the gap and enhance the prediction of SGA when ultrasound is performed remote from delivery, when the predictive accuracy of EFW is reduced.

Doppler parameters may not improve the prediction of SGA at term compared with preterm owing to differing pathophysiological processes that distinguish early‐ from late‐onset FGR. In late‐onset FGR, Doppler values are usually normal or modestly abnormal^33^ and 50–70% of SGA neonates are constitutionally small^34^ with no placental pathology^35^, ^36^ and normal Doppler parameters^37^.

Our study utilized data from routine ultrasound examinations conducted at a median GA of 36 + 4 weeks in an unselected population. Consequently, there was a higher prevalence of constitutionally small neonates and a lower prevalence of severely growth‐restricted fetuses with severe Doppler abnormalities, as these fetuses would probably have been detected and delivered before term.

### Clinical and research implications

Contemporary obstetric practice typically comprises a first‐trimester risk assessment combining maternal demographic and clinical characteristics with early ultrasound findings and serum biochemical markers to identify women at risk of placentally mediated adverse outcomes, who would benefit from heightened antepartum surveillance and pharmacological prophylaxis^38^, ^39^, ^40^, ^41^, ^42^. While these assessments are effective at predicting complications in the preterm period, the predictive accuracy for complications at term, particularly growth disorders, is poor^43^.

Term‐born neonates that are SGA are at increased risk of a range of short‐ and long‐term complications compared with those that are appropriate‐for‐gestational age^44^, ^45^, ^46^, ^47^, ^48^, ^49^, ^50^, ^51^, ^52^, ^53^, ^54^. Neonates that are SGA owing to placental insufficiency have poorer neurodevelopmental outcomes than constitutionally small neonates^55^. However, despite being considered a variant of the norm, the neurological function of constitutionally small neonates is inferior to those who are born with a birth weight > 10^th^ centile^56^.

Multiple studies demonstrating abnormal brain structure and metabolism *in utero* in small fetuses suggest that a hostile intrauterine environment and chronic hypoxia contribute to adverse neurodevelopmental outcomes as well as impaired growth, even when there is no evidence of placental disease^57^, raising the question of whether there is a neurodevelopmental benefit of earlier delivery for these fetuses.

Of equal importance is the strong association between unidentified SGA and perinatal death^58^. More than half of stillbirths are SGA, the majority of which have no evidence of placental insufficiency prenatally^59^, ^60^, ^61^. Antenatal detection and implementation of antenatal surveillance and indicated delivery can reduce the risk of perinatal death^59^, ^62^. As a result, efforts have been directed towards improving the detection of fetuses at risk of SGA, particularly at term^63^.

We propose that introducing a late third‐trimester routine ultrasound examination would provide an opportunity to reassess risk as the pregnancy enters its final stage. Performing such a risk assessment at this stage of pregnancy is particularly important considering that the cost of intervention (i.e. timed delivery), in terms of neonatal morbidity, is relatively low^21^, ^22^ and the prospective risk of stillbirth increases with advancing GA after term^20^, particularly for SGA neonates^1^.

Our findings reveal that a late third‐trimester examination accurately predicts fetuses at risk of being SGA and having poor outcomes. Our finding that the incorporation of Doppler indices did not enhance the predictive accuracy is consistent with the findings of other studies and is valuable for resource‐limited settings, where sonographic expertise may be scarce and fetal Doppler evaluation less available.

### Strengths and limitations

The strengths of our study derive from including a large population of unselected low‐risk patients who underwent a comprehensive sonographic evaluation in the late third trimester within a short interval before birth by trained operators in a real‐life setting. This allowed us to evaluate and compare the performance of a range of variables including maternal characteristics, standard fetal biometry and Doppler parameters (UA‐PI, MCA‐PI, CPR and UtA‐PI), widely considered markers of placental insufficiency for the prediction of adverse outcomes in pregnancies that have reached term, and adds important data that could aid in the assessment and management of pregnancies at term.

This study was limited by its retrospective nature and the associated treatment selection bias. Managing obstetricians were not blinded to the results of the sonographic evaluation, therefore, according to our local protocol, pregnancies at risk for SGA or with abnormal fetal Doppler were more likely to have undergone elective delivery. Indeed, the rate of induction of labor in our study was 57% in fetuses that were SGA compared with 35% in those that were not. This finding is likely to have attenuated the predictive performance of Doppler parameters, as pregnancies deemed to be at risk were likely to have been delivered earlier, prior to the onset of Doppler abnormalities. Furthermore, perinatal outcomes that are of most significance to parents, namely stillbirth and neonatal death, were rare in this cohort, therefore the study lacked sufficient power to detect an effect on these outcomes.

### Conclusions

Sonographic fetal biometry evaluation in the late third trimester can predict the delivery of an SGA or FGR neonate, including those at risk of adverse perinatal outcomes. The addition of Doppler parameters to fetal biometry did not improve the prediction of small neonates.

## Supporting information


**Figure S1–S13** Calibration plots for the prediction of small‐for‐gestational age < 5^th^ centile according to: maternal characteristics (Figure S1); estimated fetal weight centile (Figure S2); abdominal circumference centile (Figure S3); umbilical artery pulsatility index (Figure S4); middle cerebral artery pulsatility index (Figure S5); cerebroplacental ratio (Figure S6); uterine artery pulsatility index (Figure S7); estimated fetal weight centile and umbilical artery pulsatility index (Figure S8); estimated fetal weight centile and cerebroplacental ratio (Figure S9); estimated fetal weight centile, umbilical artery pulsatility index and middle cerebral artery pulsatility index (Figure S10); estimated fetal weight centile, umbilical artery pulsatility index and uterine artery pulsatility index (Figure S11); estimated fetal weight centile, uterine artery pulsatility index and cerebroplacental ratio (Figure S12); estimated fetal weight centile, middle cerebral artery pulsatility index and uterine artery pulsatility index (Figure S13).
**Figure S14–S26** Calibration plots for the prediction of fetal growth restriction according to: maternal characteristics (Figure S14); estimated fetal weight centile (Figure S15); abdominal circumference centile (Figure S16); umbilical artery pulsatility index (Figure S17); middle cerebral artery pulsatility index (Figure S18); cerebroplacental ratio (Figure S19); uterine artery pulsatility index (Figure S20); estimated fetal weight centile and umbilical artery pulsatility index (Figure S21); estimated fetal weight centile and cerebroplacental ratio (Figure S22); estimated fetal weight centile, umbilical artery pulsatility index and middle cerebral artery pulsatility index (Figure S23); estimated fetal weight centile, umbilical artery pulsatility index and uterine artery pulsatility index (Figure S24); estimated fetal weight centile, cerebroplacental ratio and uterine artery pulsatility index (Figure S25); estimated fetal weight centile, middle cerebral artery pulsatility index and uterine artery pulsatility index (Figure S26).

## Data Availability

The data that support the findings of this study are available from the corresponding author upon reasonable request.
