# Supplementary material for: Prediction of small‐for‐gestational age and fetal growth restriction at routine ultrasound examination at 35–37 weeks' gestation
Source: Ultrasound Obstet Gynecol. 2025 Apr 26;65(6):761–70. doi: 10.1002/uog.29223 (PMC12127726; doi:10.1002/uog.29223)

**Supplementary Figures.**

**Supplementary Figure 1.** Calibration plot for the prediction of SGA <5^th^ centile according to maternal characteristics.


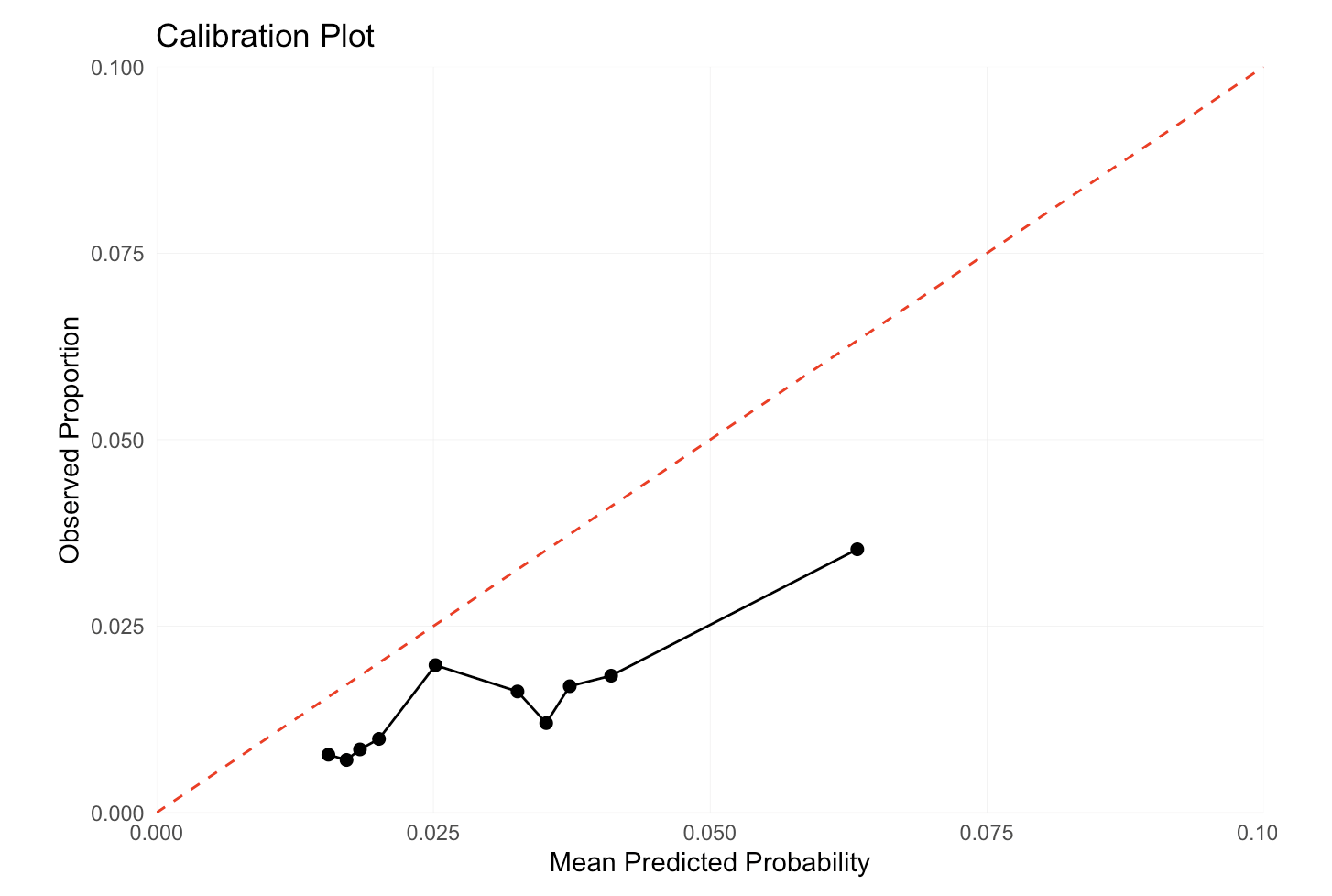


**Supplementary Figure 2.** Calibration plot for the prediction of SGA <5^th^ centile according to EFW centile


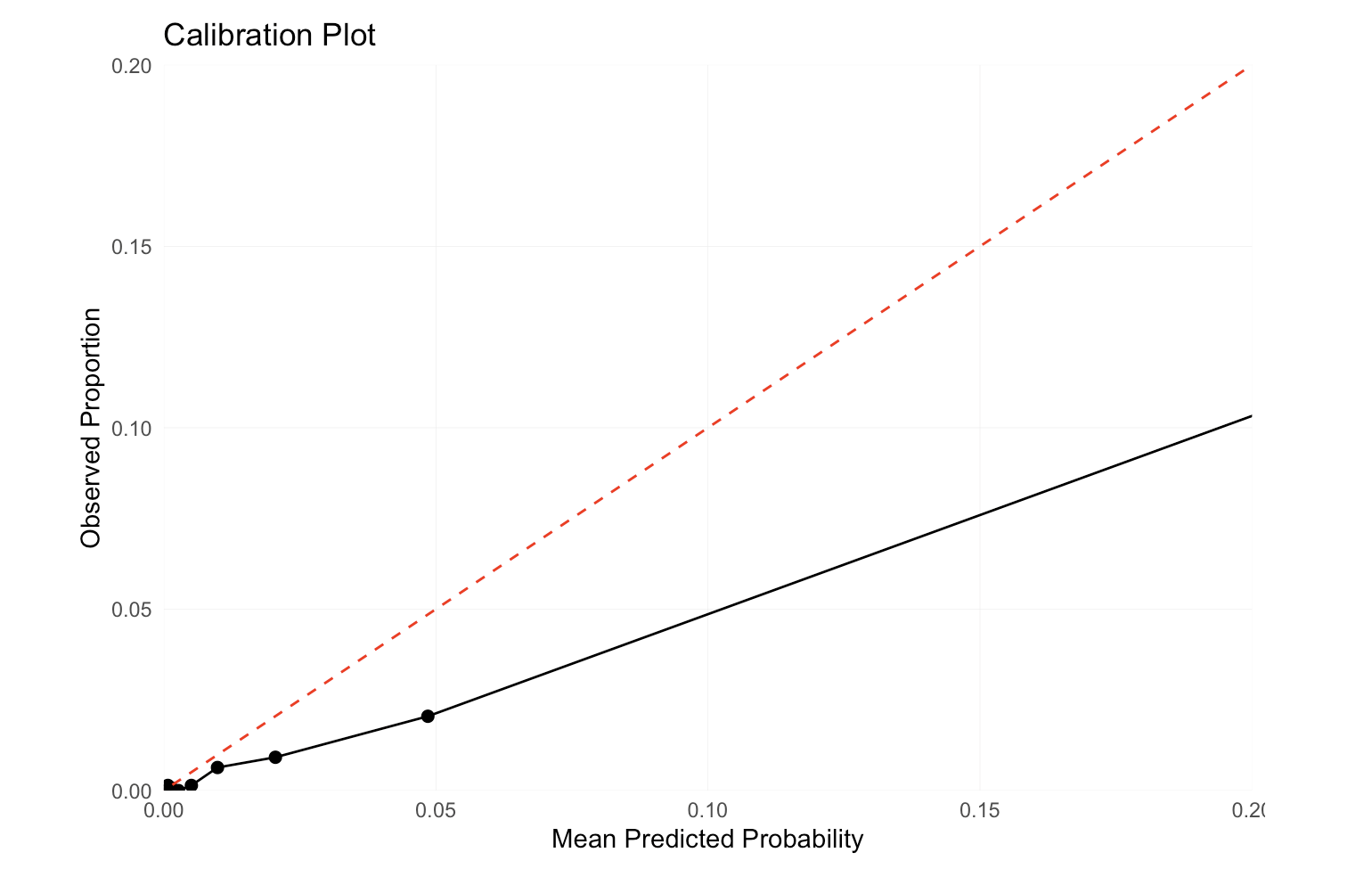


**Supplementary Figure 3.** Calibration plot for the prediction of SGA <5^th^ centile according to AC centile


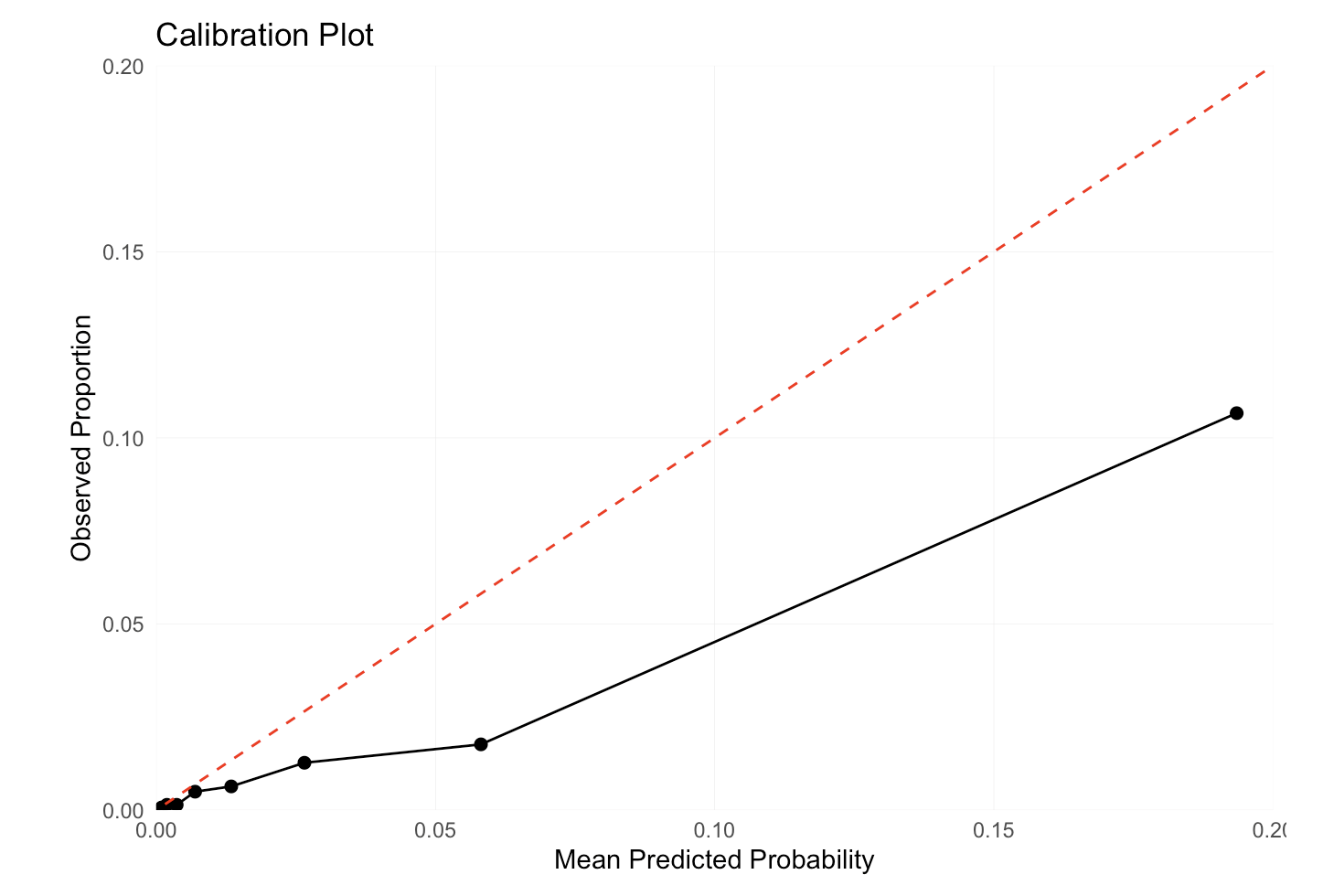


**Supplementary Figure 4.** Calibration plot for the prediction of SGA <5^th^ centile according to Umbilical Artery PI


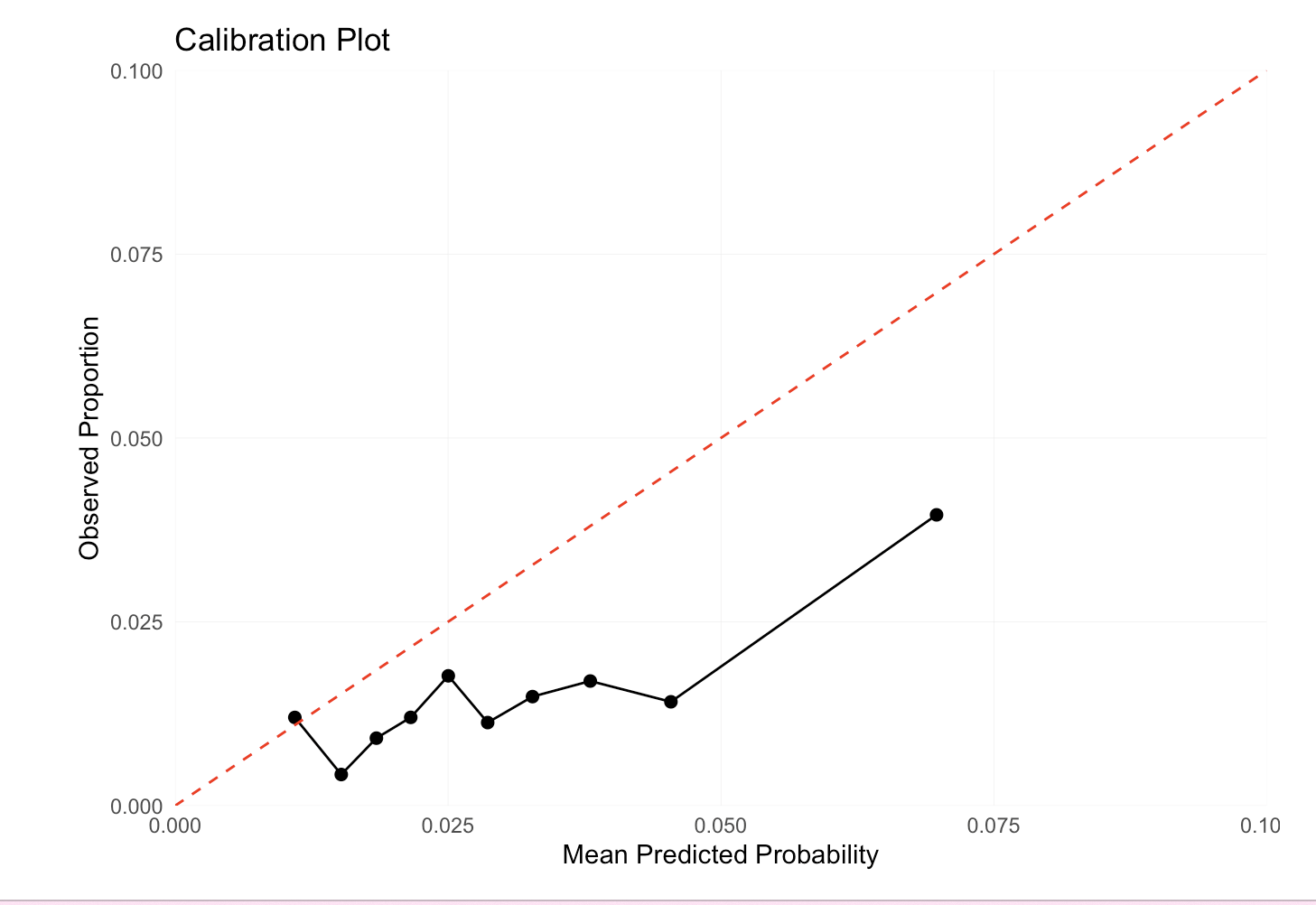


**Supplementary Figure 5.** Calibration plot for the prediction of SGA <5^th^ centile according to the Middle Cerebral Artery PI


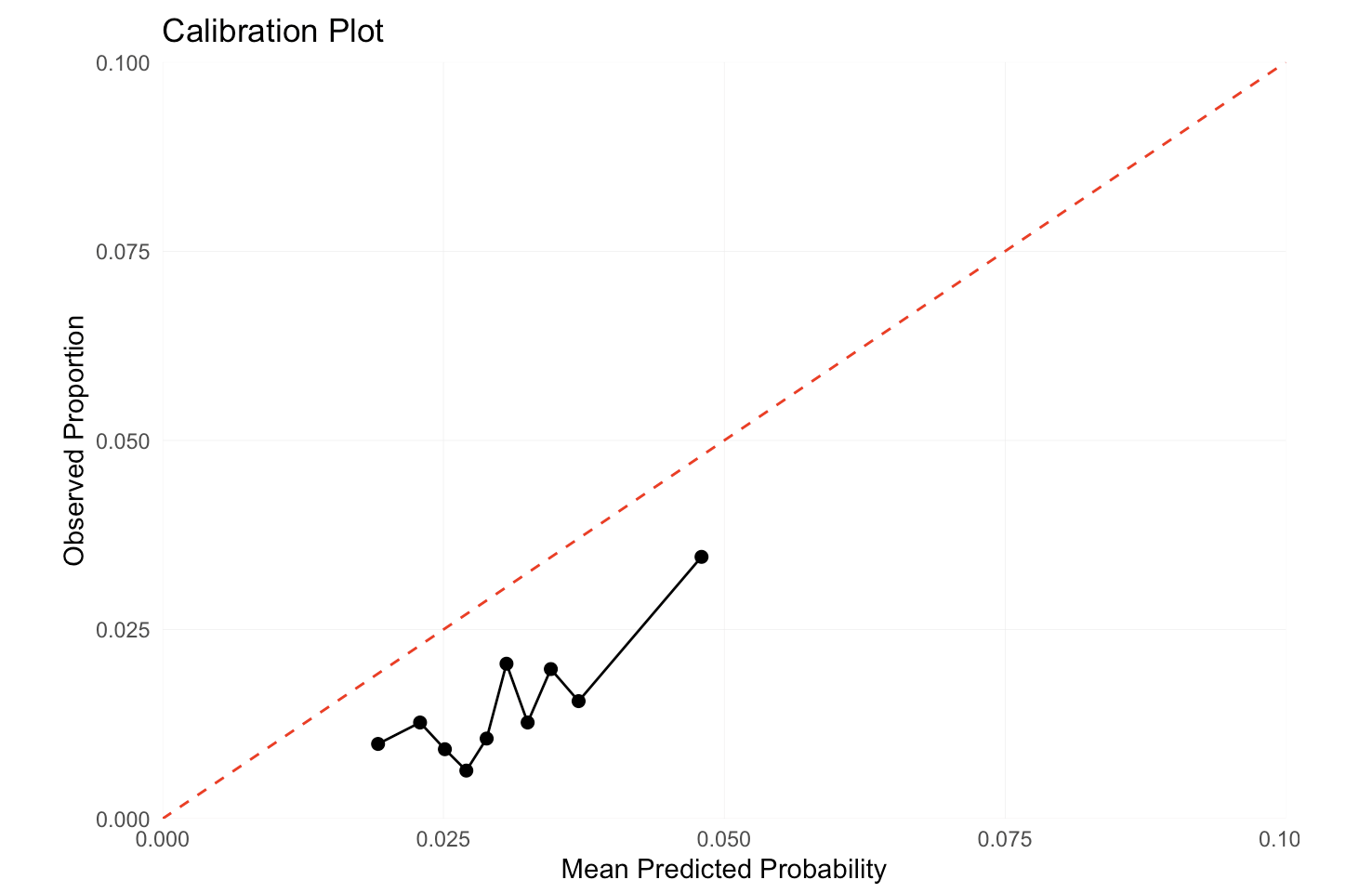


**Supplementary Figure 6.** Calibration plot for the prediction of SGA <5^th^ centile according to the Cerebroplacental ratio


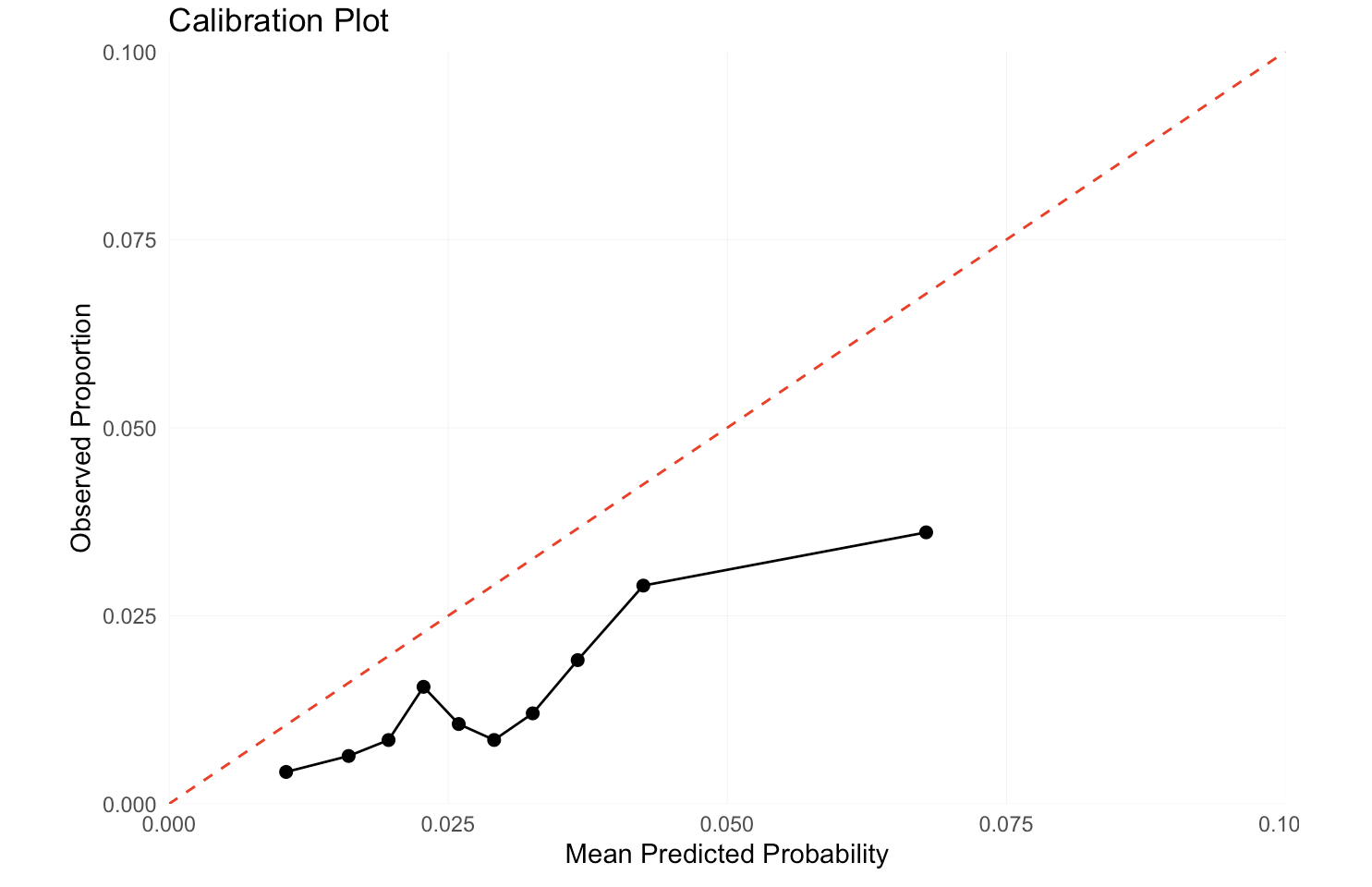


**Supplementary Figure 7.** Calibration plot for the prediction of SGA <5^th^ centile according to the Uterine Artery PI


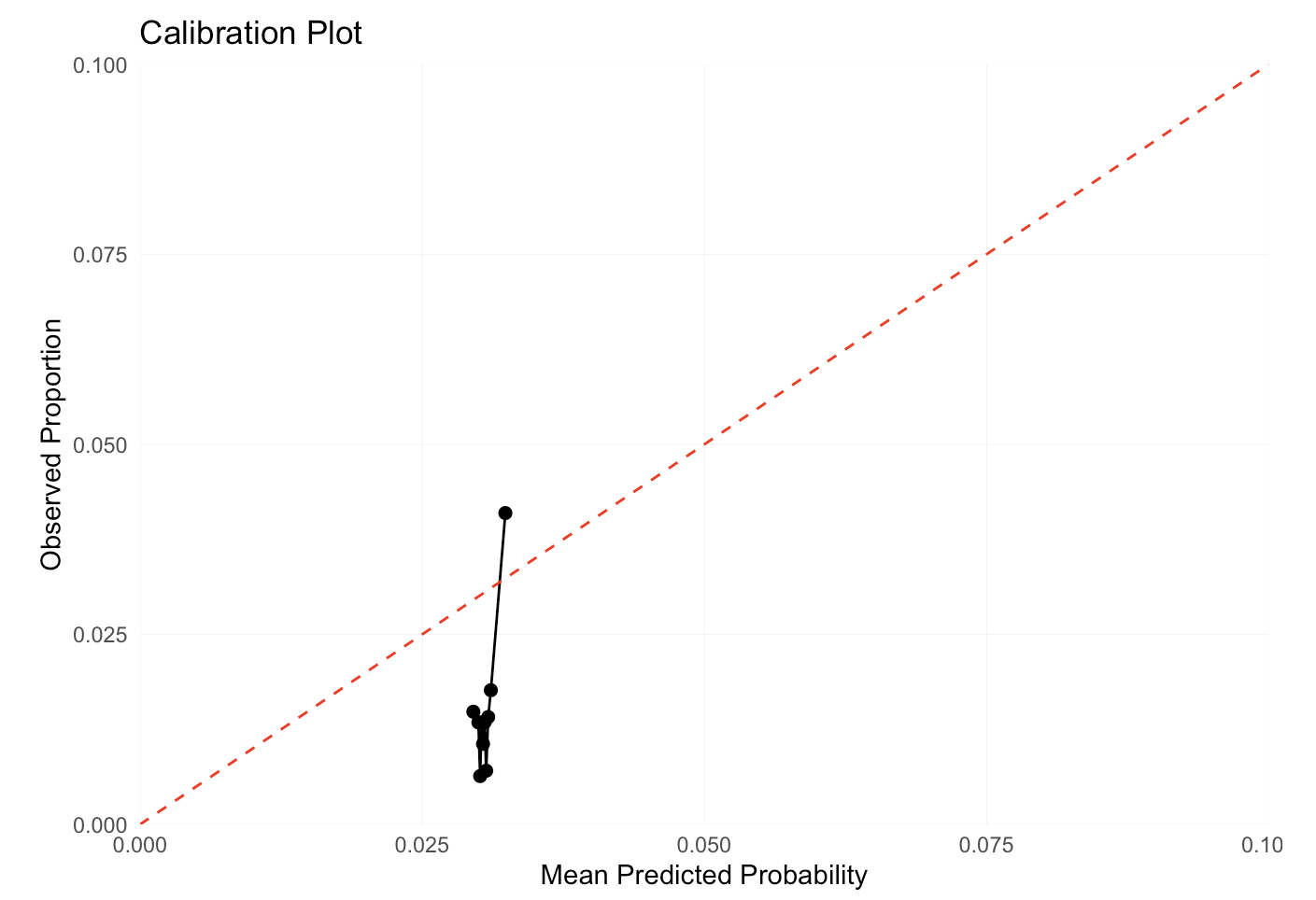


**Supplementary Figure 8.** Calibration plot for the prediction of SGA <5^th^ centile according to the EFW Centile and Umbilical Artery PI


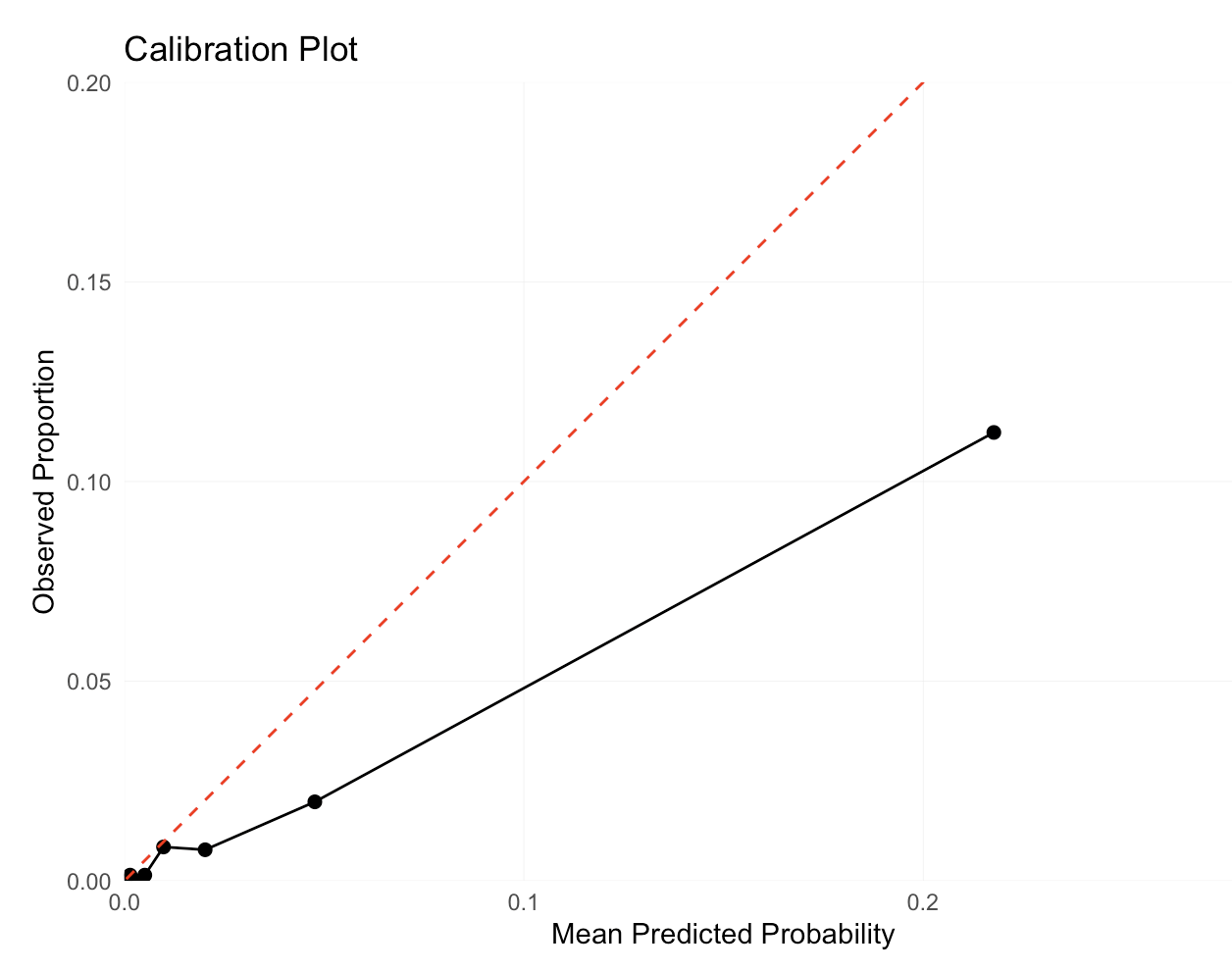


**Supplementary Figure 9.** Calibration plot for the prediction of SGA <5^th^ centile according to the EFW Centile and CPR


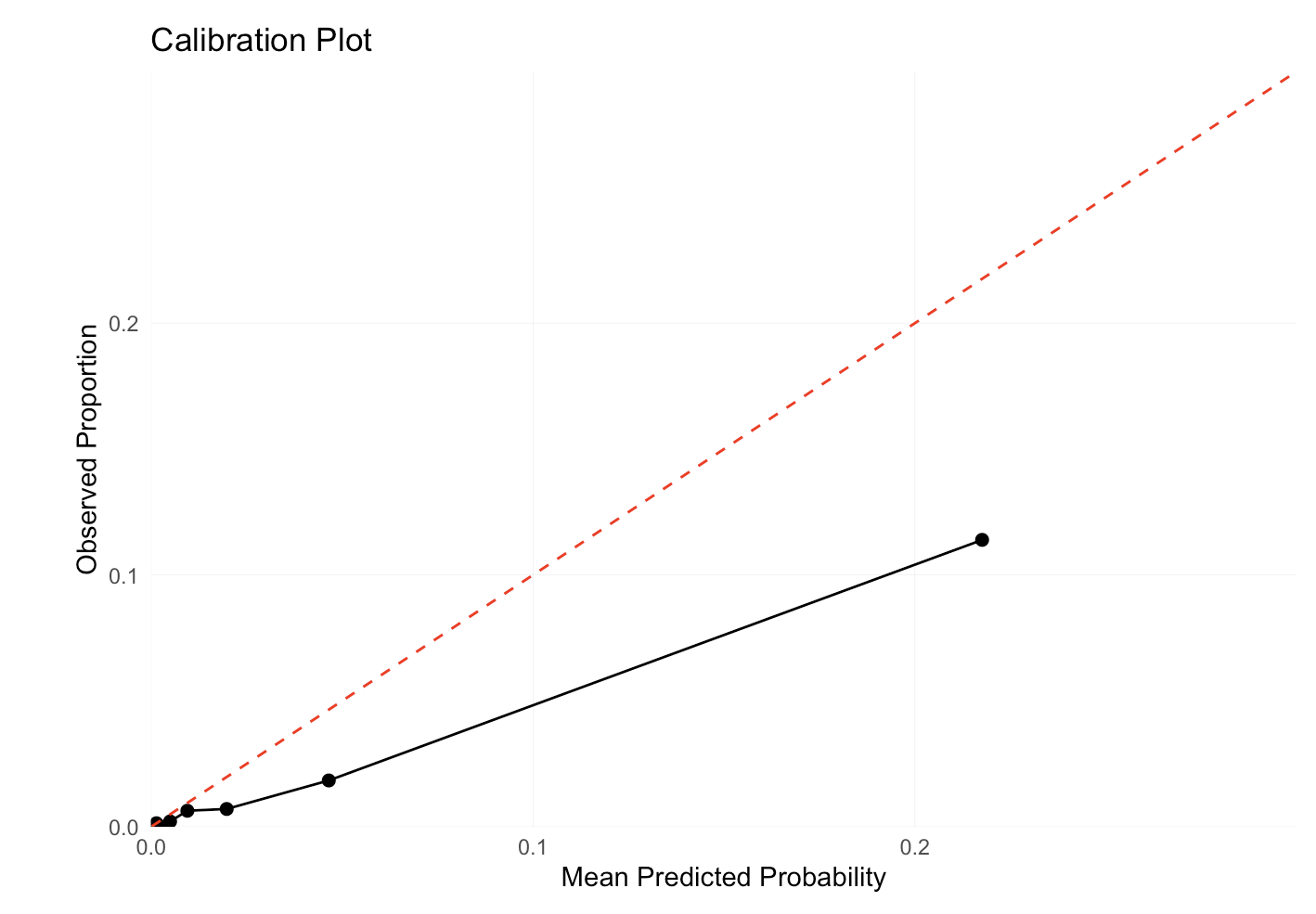


**Supplementary Figure 10.** Calibration plot for the prediction of SGA<5^th^ centile according to EFW centile, Umbilical Artery PI and MCA PI.

**
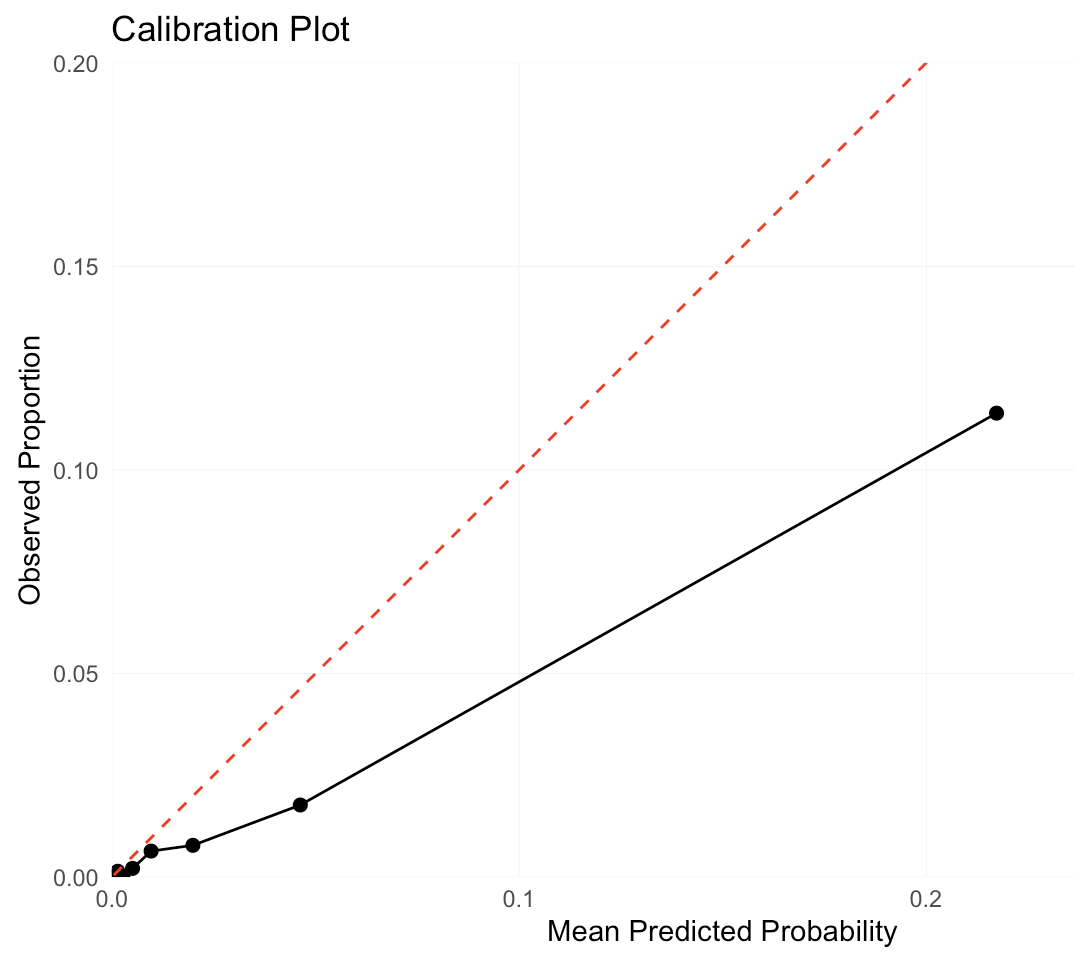
**

**Supplementary Figure 11.** Calibration plot for the prediction of SGA<5^th^ centile according to EFW centile, Umbilical Artery PI and Uterine Artery PI.

**
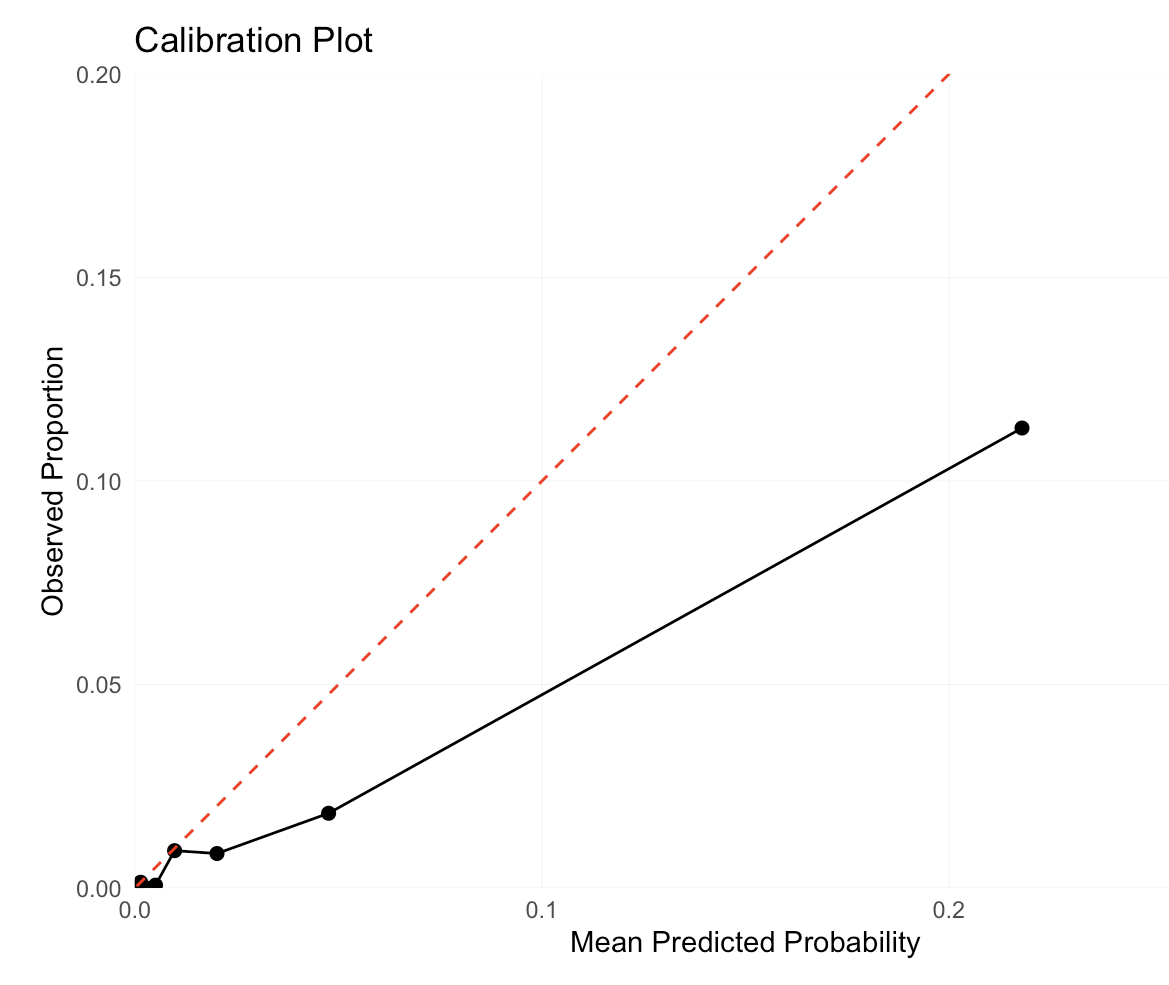
**

**Supplementary Figure 12.** Calibration plot for the prediction of SGA<5^th^ centile according to EFW centile, Uterine Artery PI and CPR.


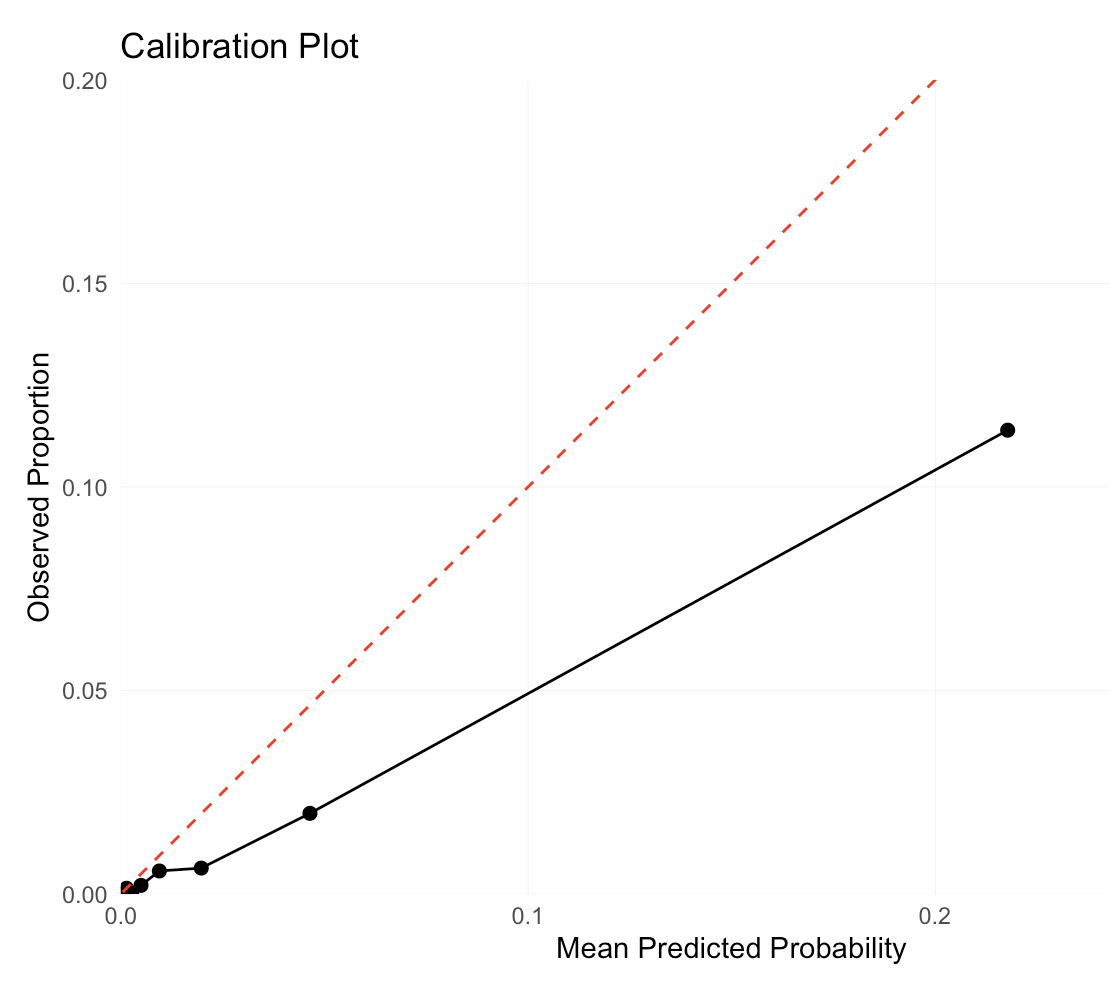


**Supplementary Figure 13.** Calibration plot for the prediction of SGA<5^th^ centile according to EFW centile, MCA PI and Uterine Artery PI.


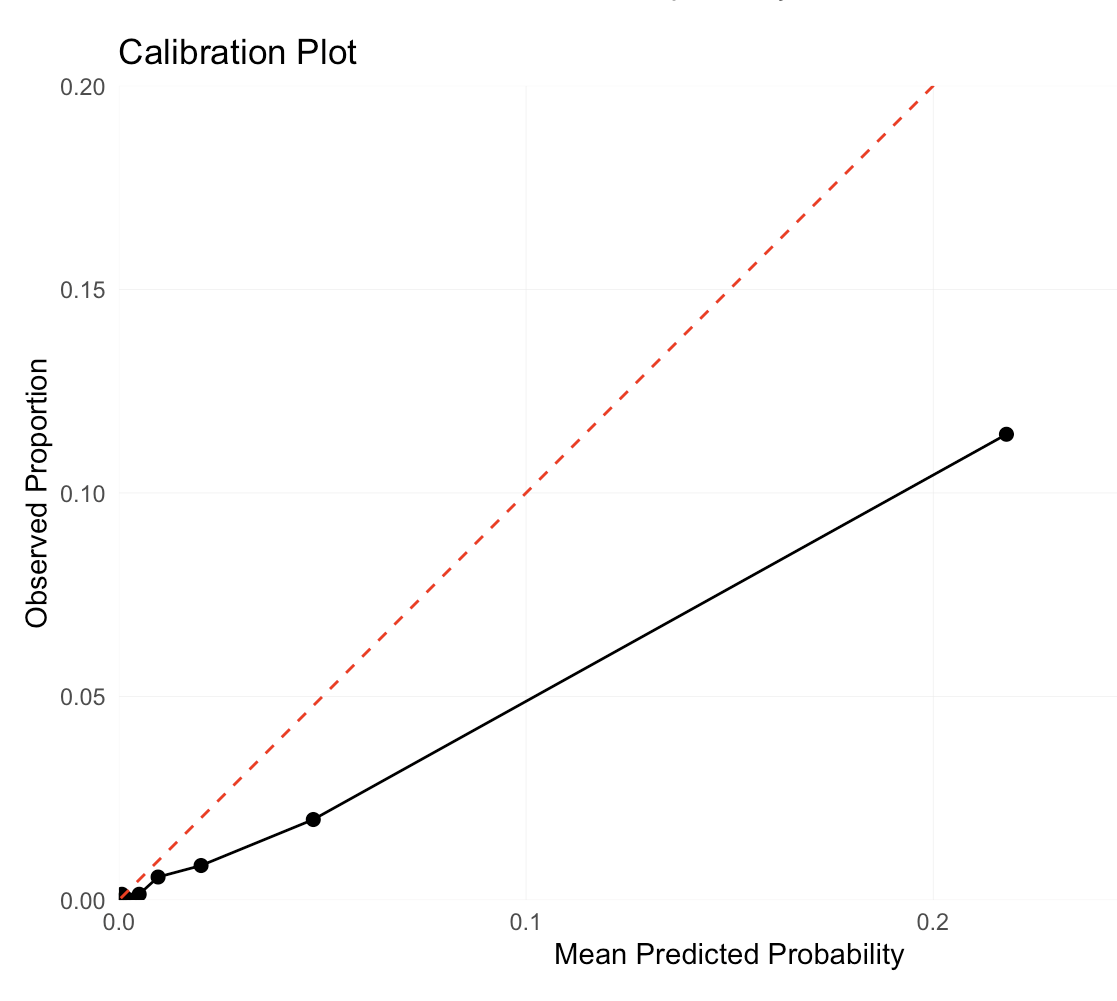


**Supplementary Figure 14.** Calibration plot for the prediction of FGR according to maternal characteristics.


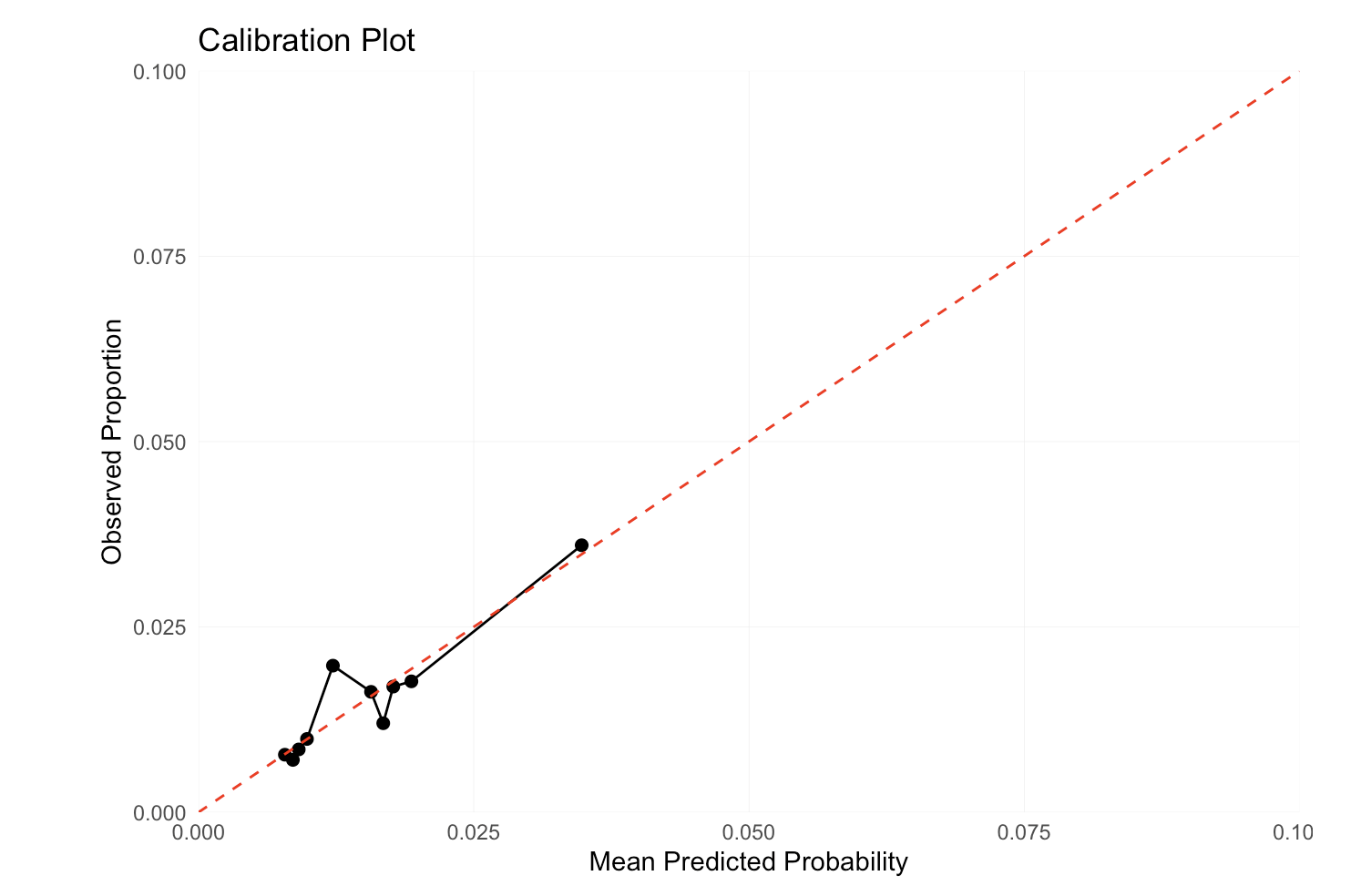


**Supplementary Figure 15.** Calibration plot for the prediction FGR according to EFW centile


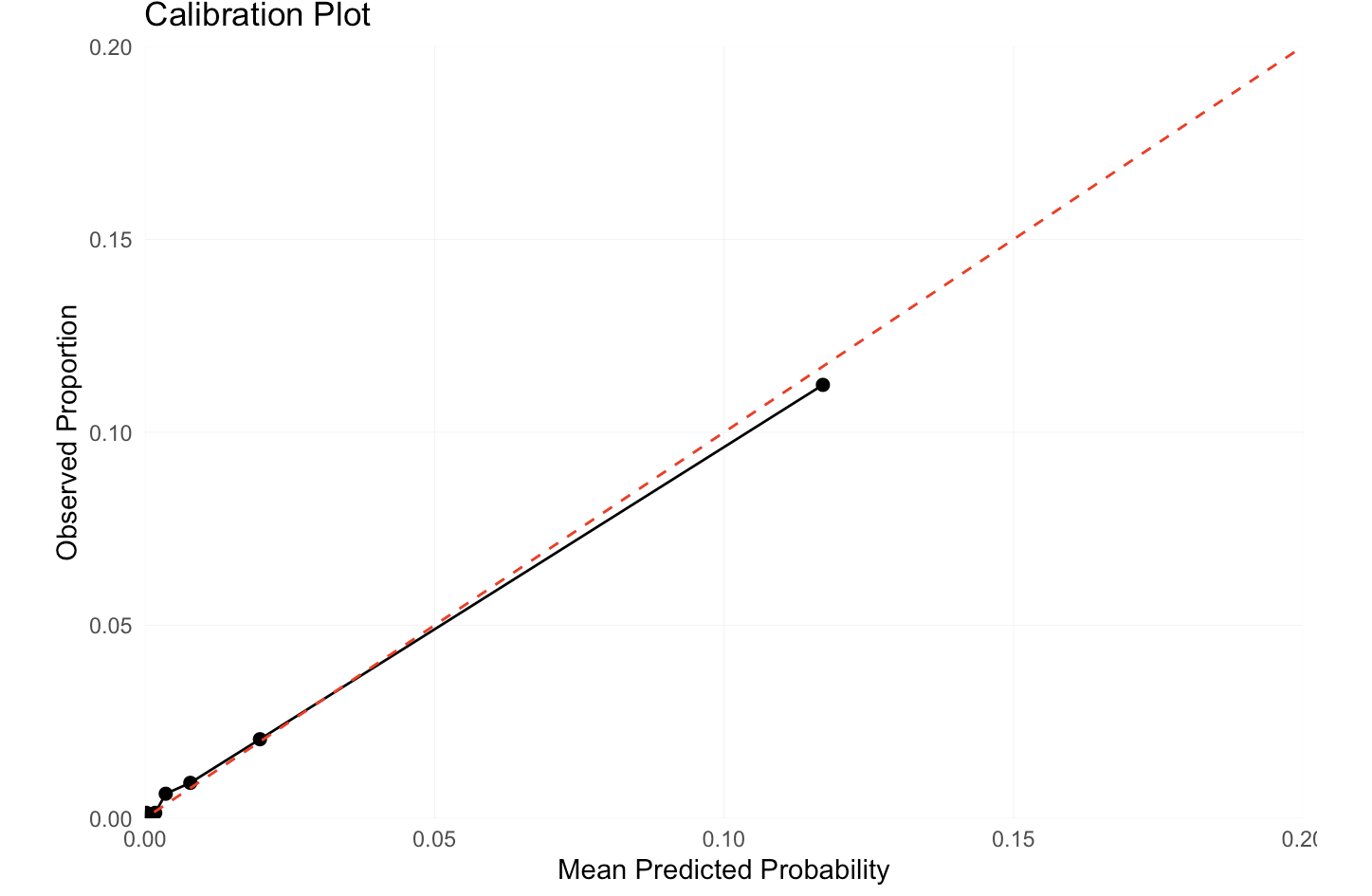


**Supplementary Figure 16.** Calibration plot for the prediction of FGR according to AC centile


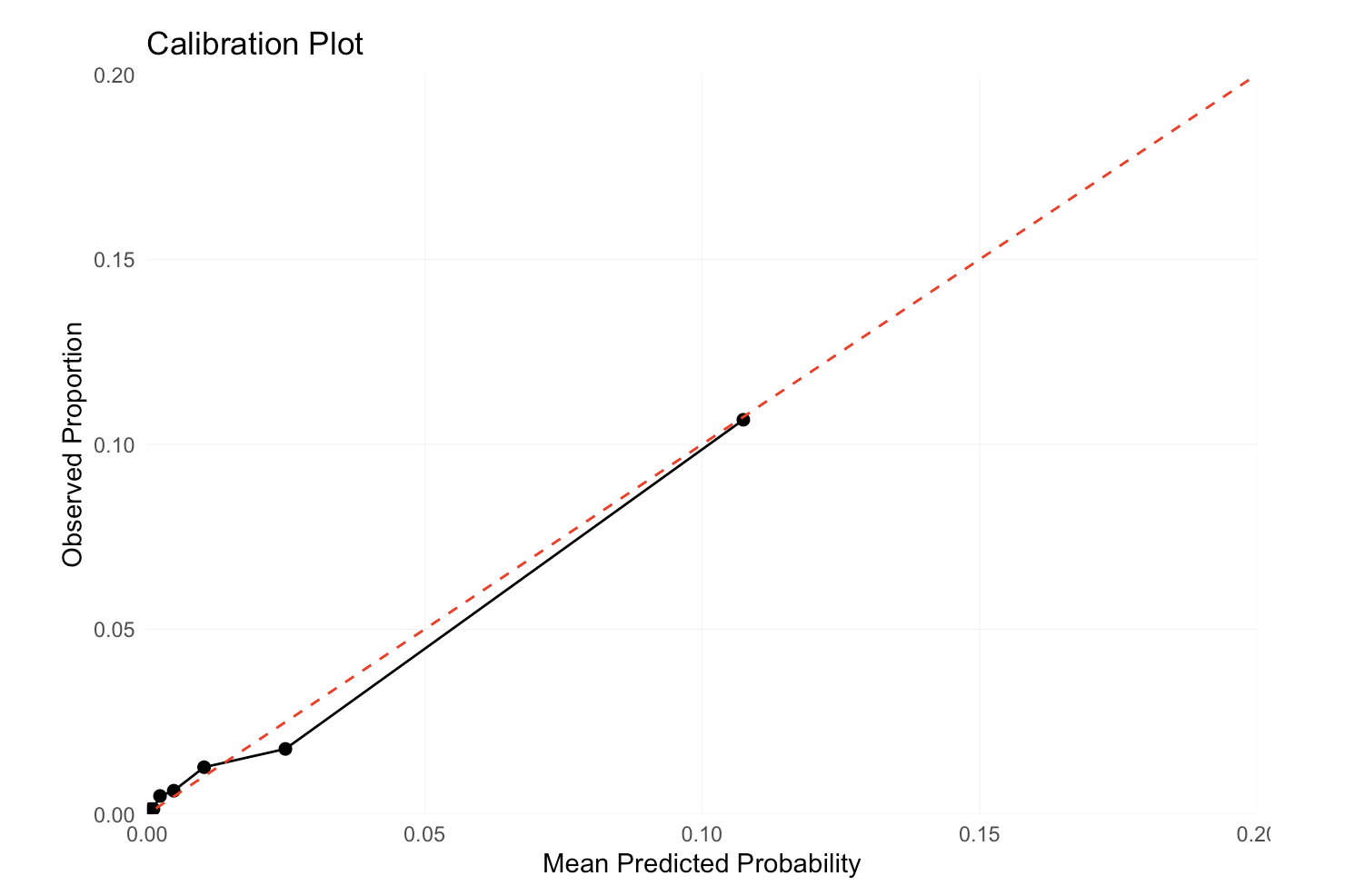


**Supplementary Figure 17**. Calibration plot for the prediction of FGR according to Umbilical Artery PI


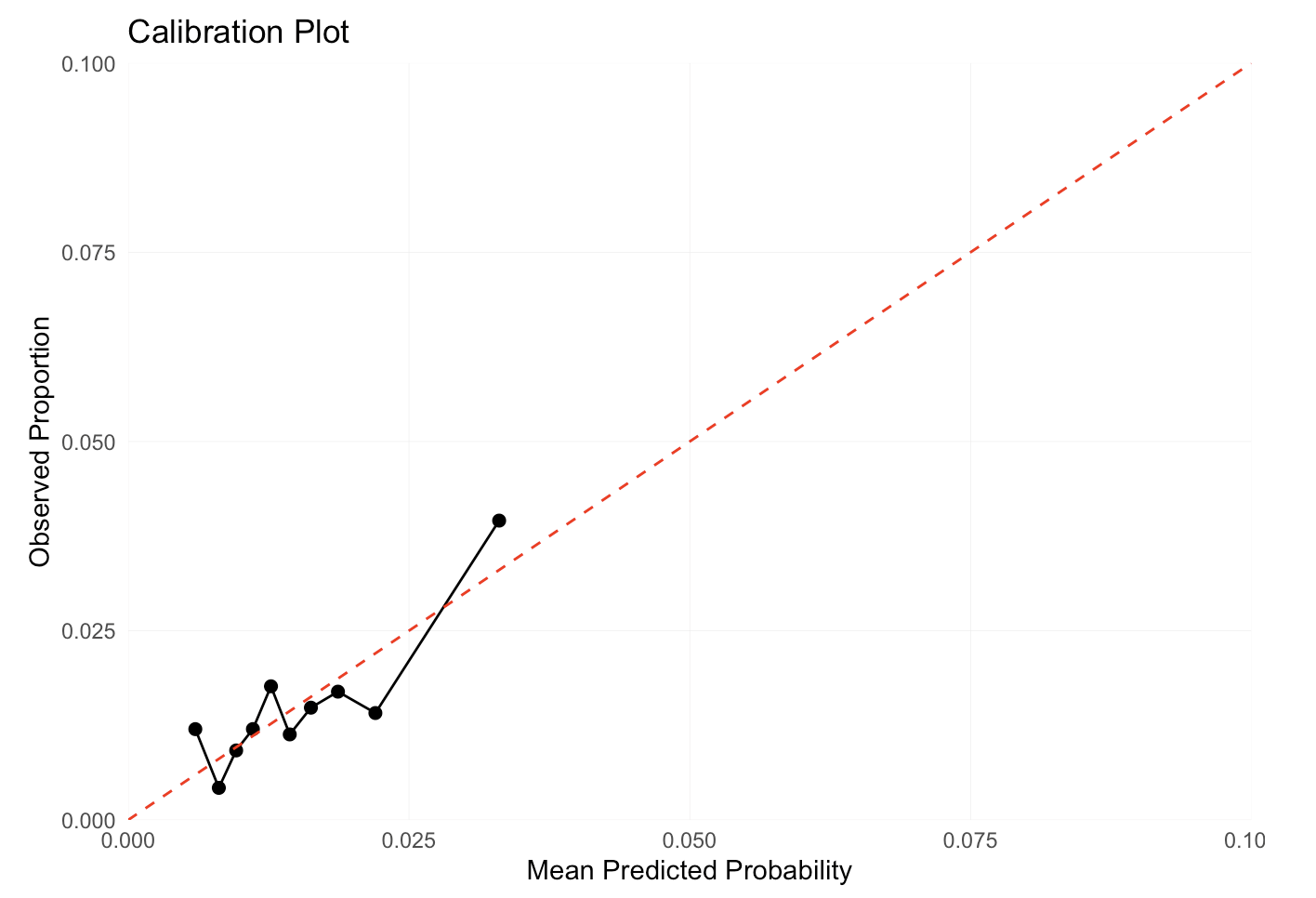


**Supplementary Figure 18.** Calibration plot for the prediction of FGR according to the Middle Cerebral Artery PI


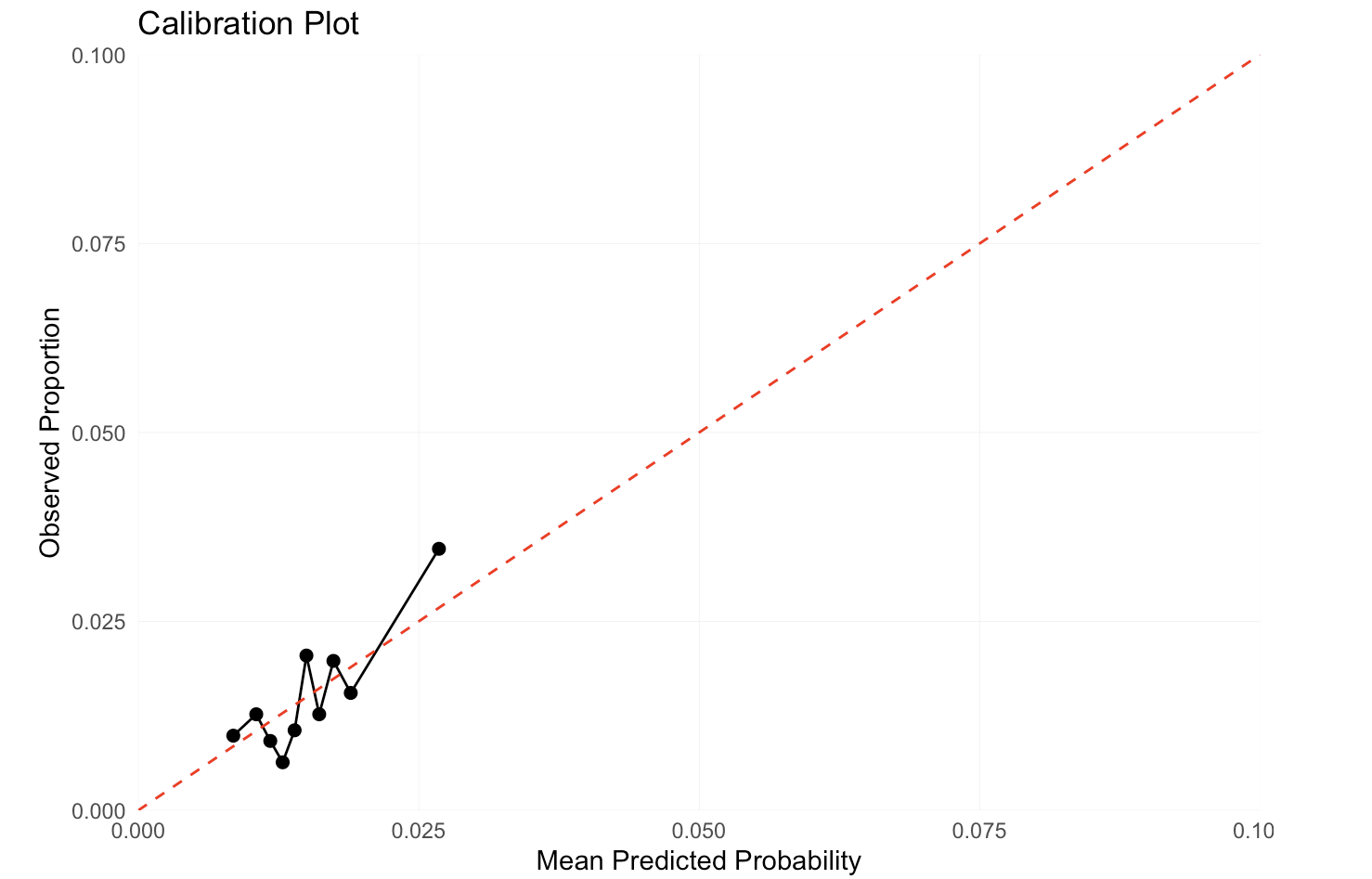


**Supplementary Figure 19.** Calibration plot for the prediction of FGR according to the Cerebroplacental ratio


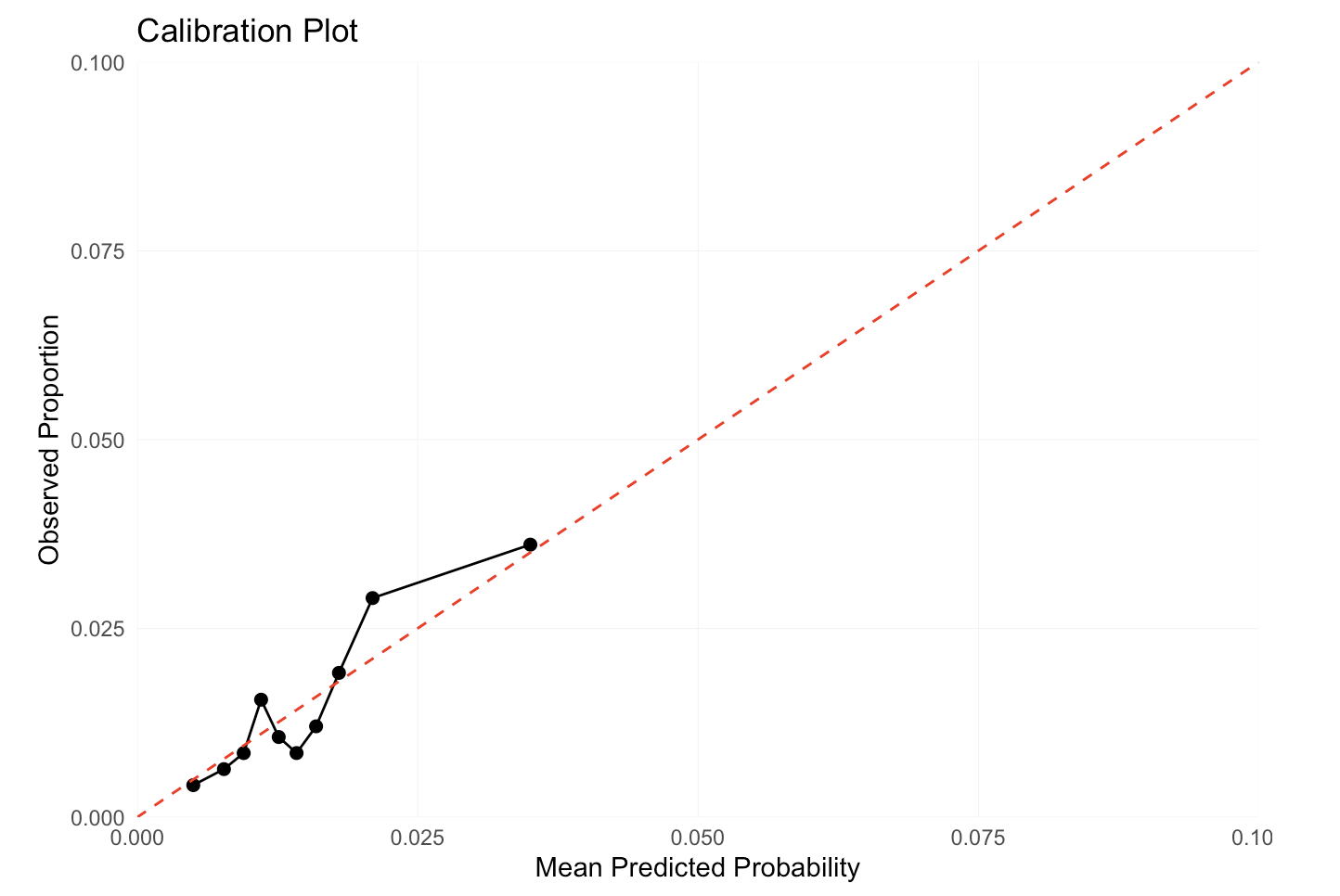


**Supplementary Figure 20.** Calibration plot for the prediction of FGR according to the Uterine Artery PI


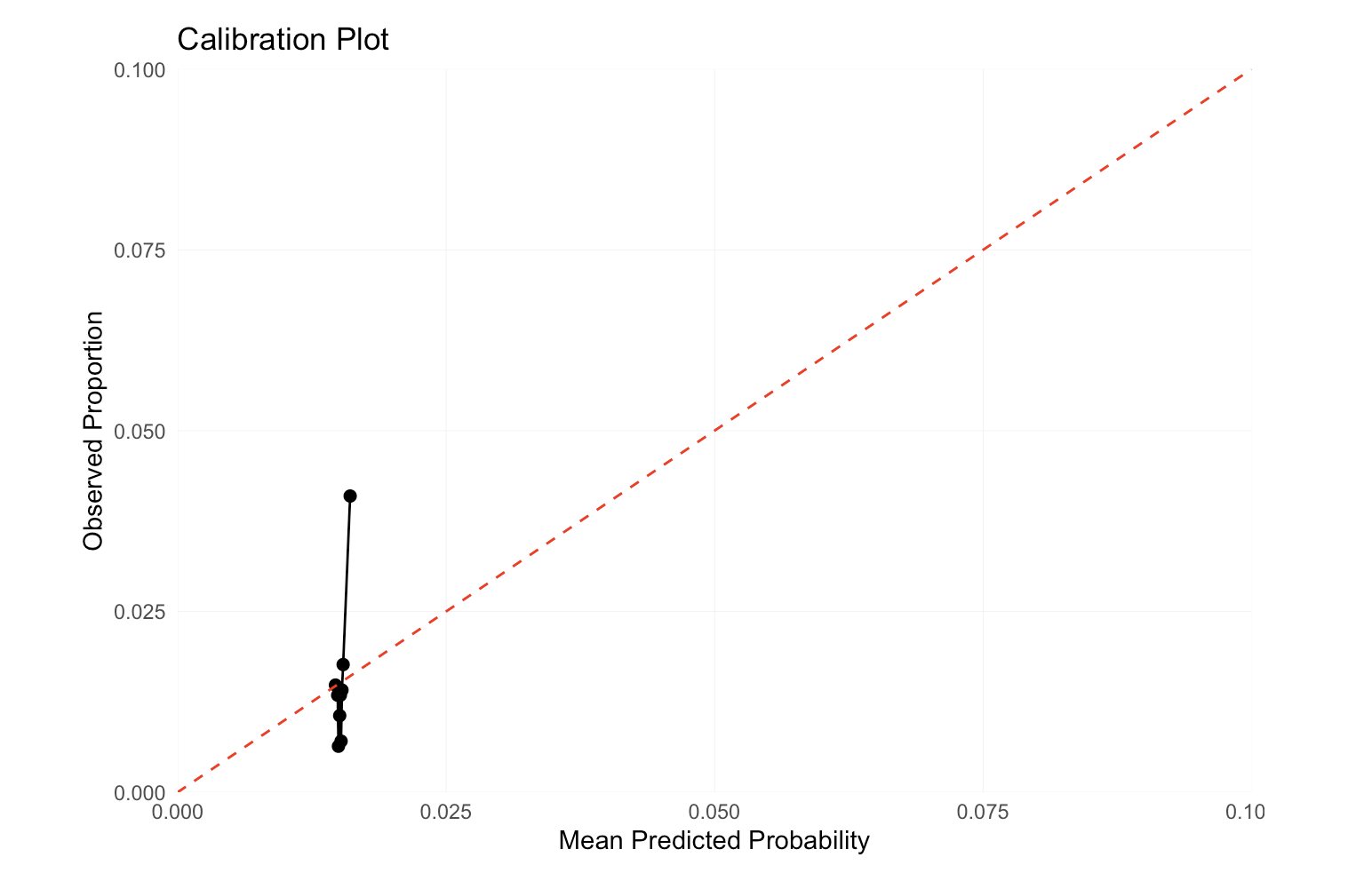


**Supplementary Figure 21.** Calibration plot for the prediction of FGR according to the EFW Centile and Umbilical Artery PI.


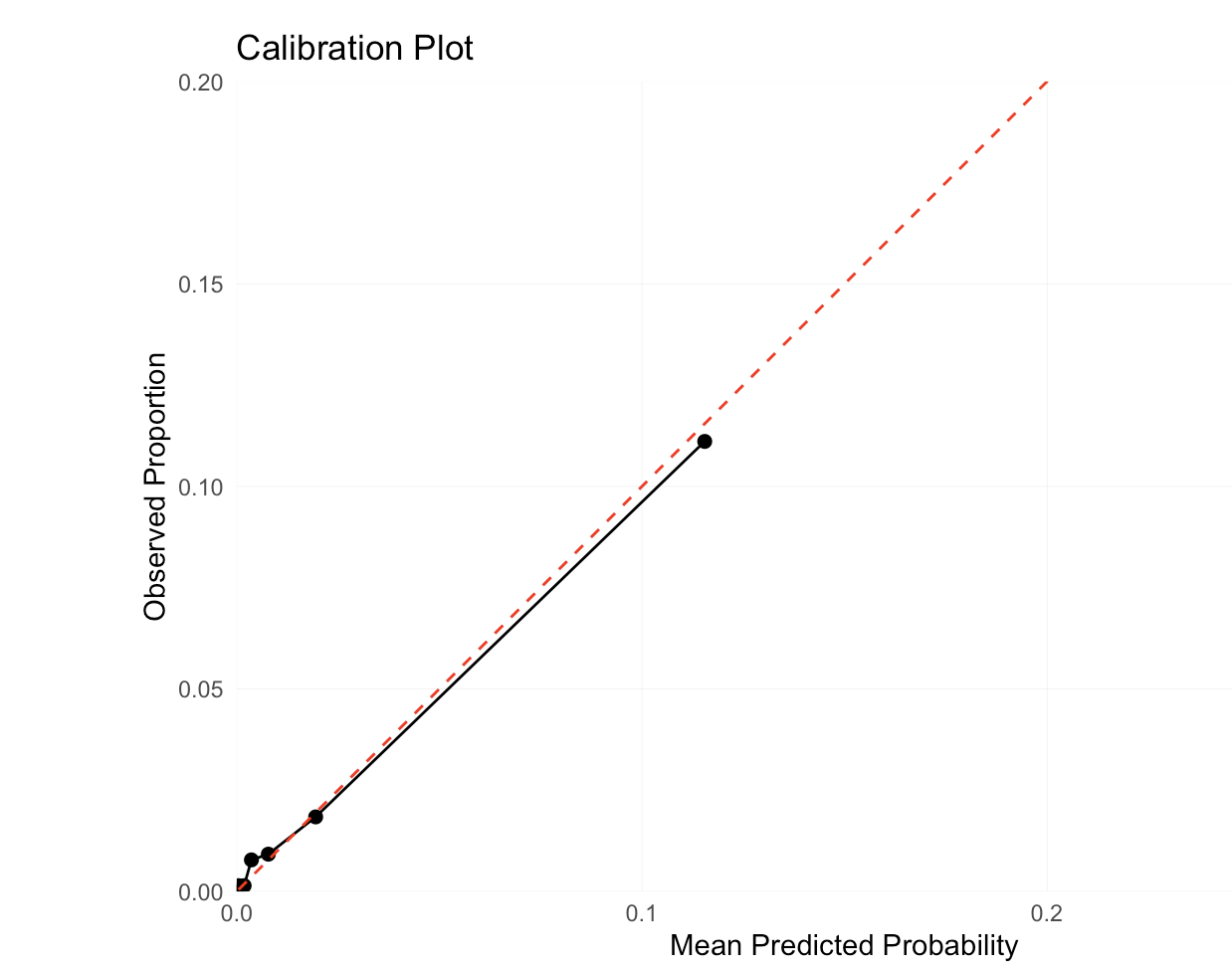


**Supplementary Figure 22.** Calibration plot for the prediction of FGR according to the EFW Centile and CPR


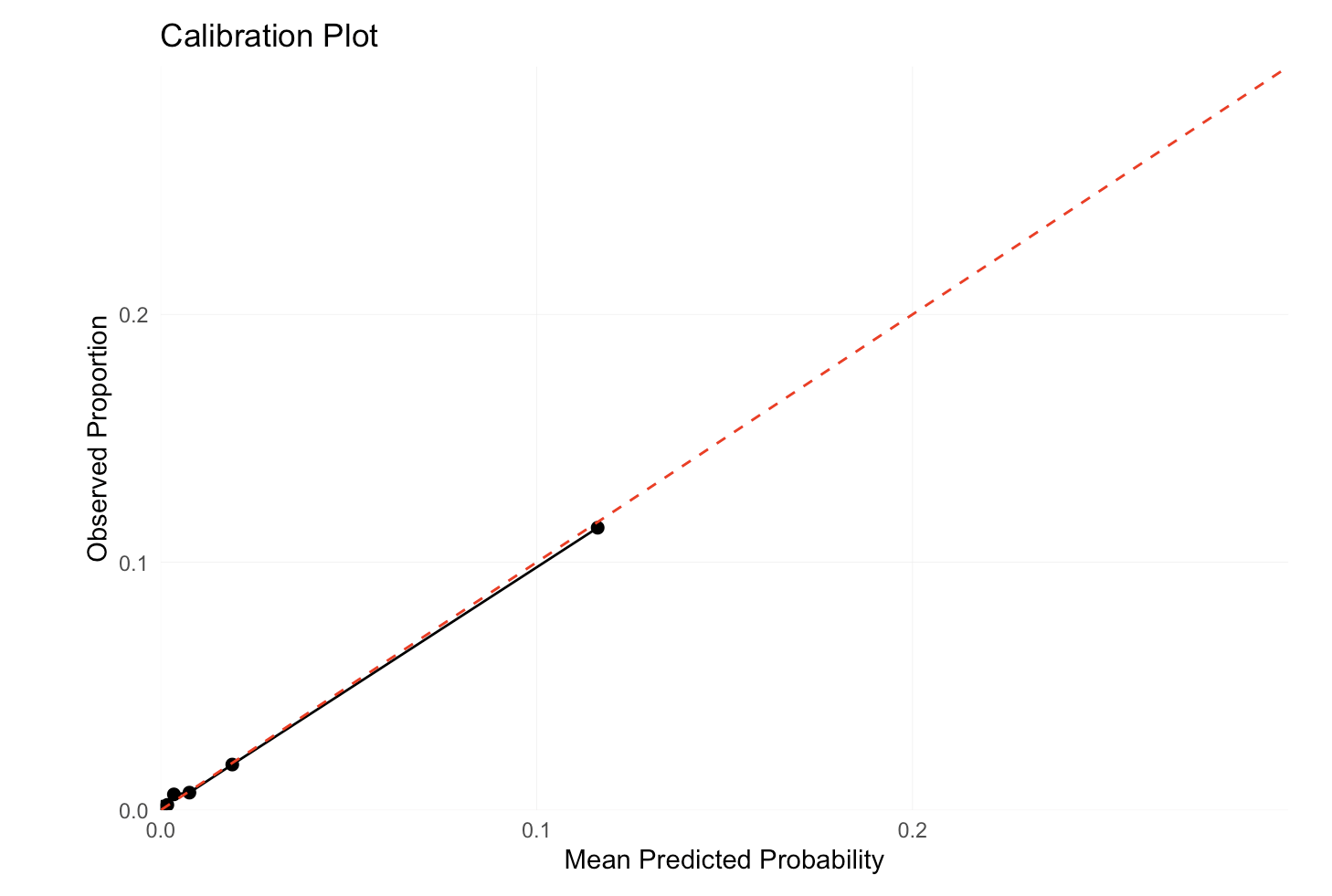


**Supplementary Figure 23.** Calibration plot for the prediction of FGR according to the EFW Centile, Umbilical Artery PI and MCA PI.


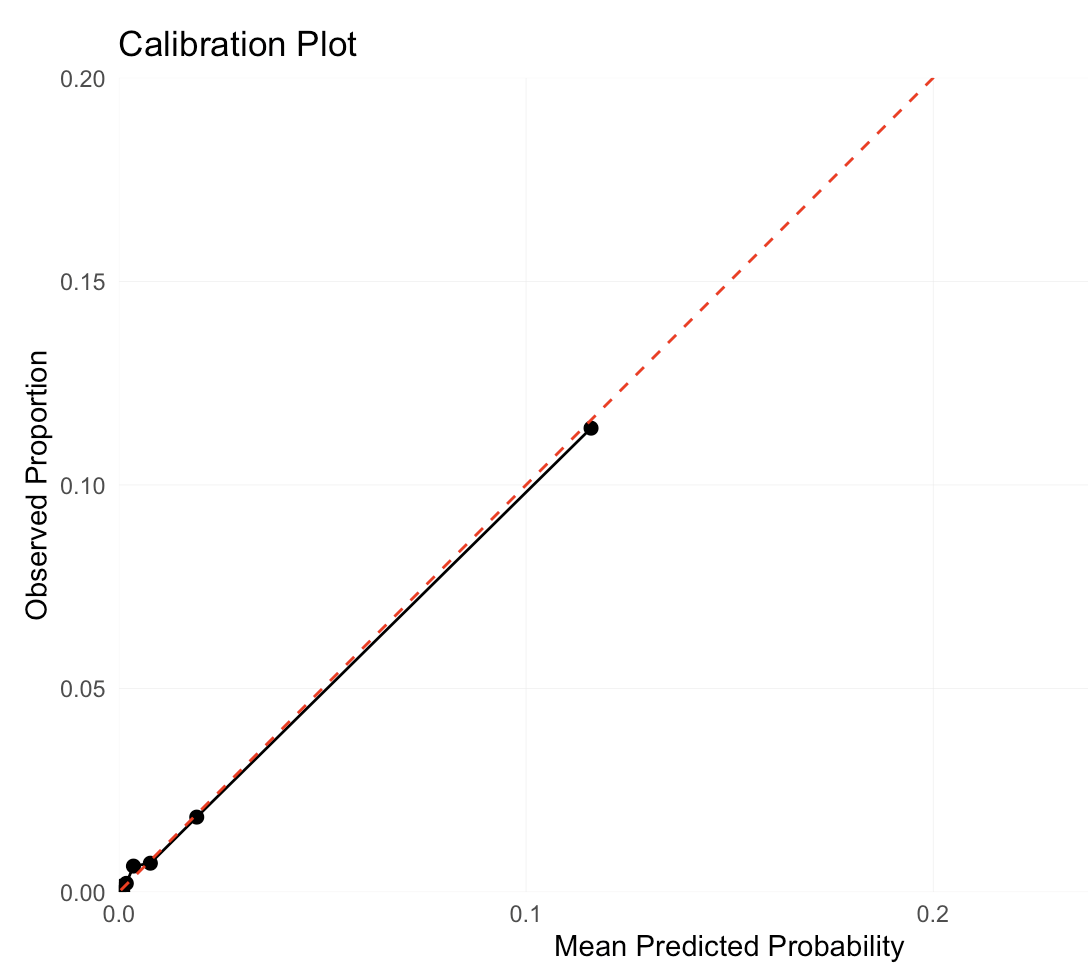


**Supplementary Figure 24.** Calibration plot for the prediction of FGR according to the EFW Centile, Umbilical Artery PI and Uterine Artery PI.


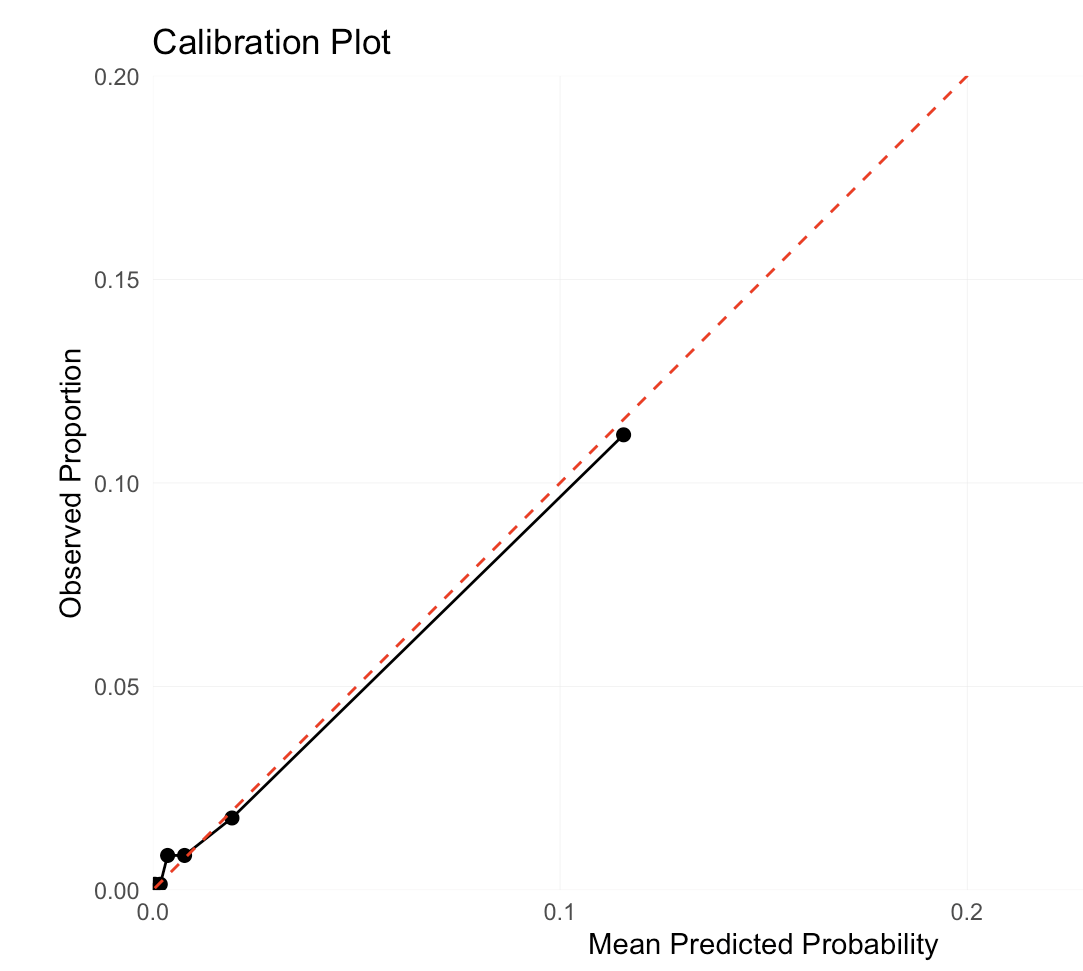


**Supplementary Figure 25.** Calibration plot for the prediction of FGR according to the EFW Centile, CPR and UtA PI


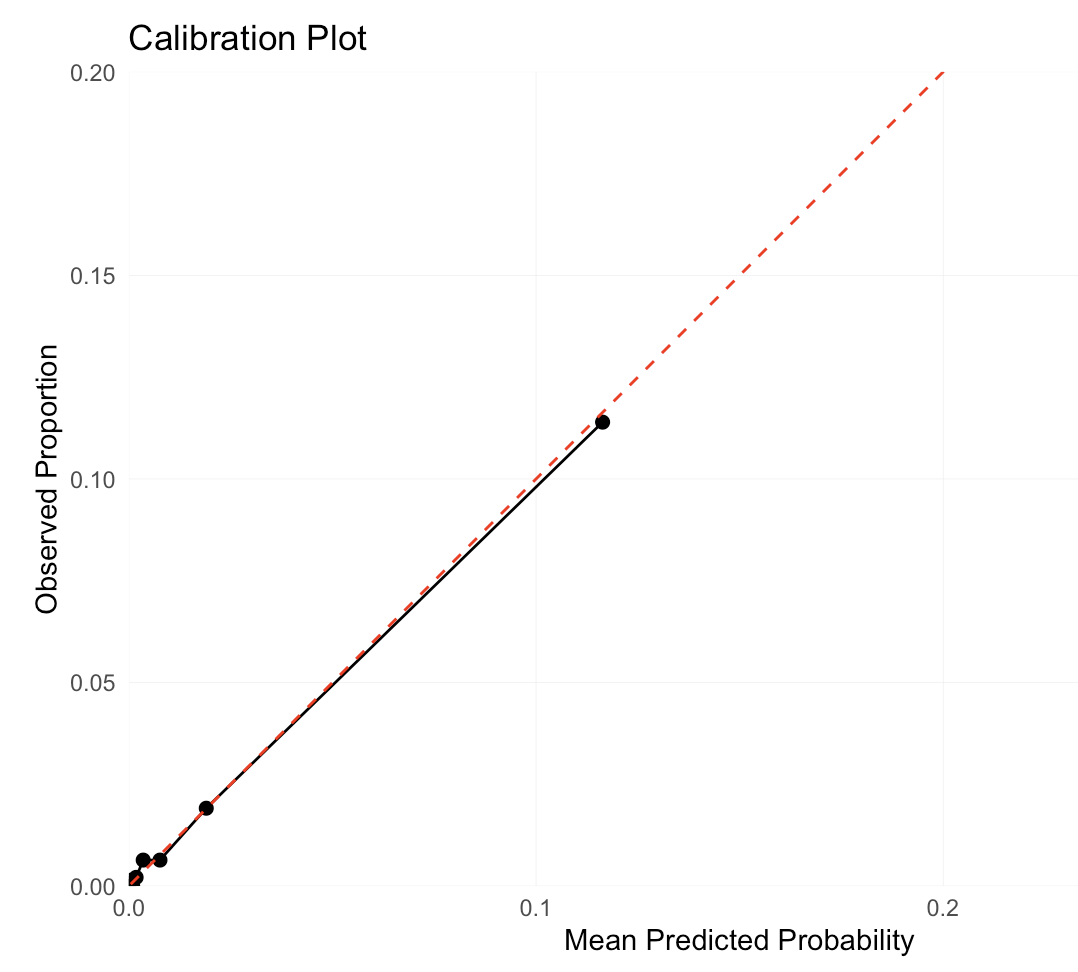


**Supplementary Figure 26.** Calibration plot for the prediction of FGR according to the EFW Centile, MCA PI and Uterine Artery PI.


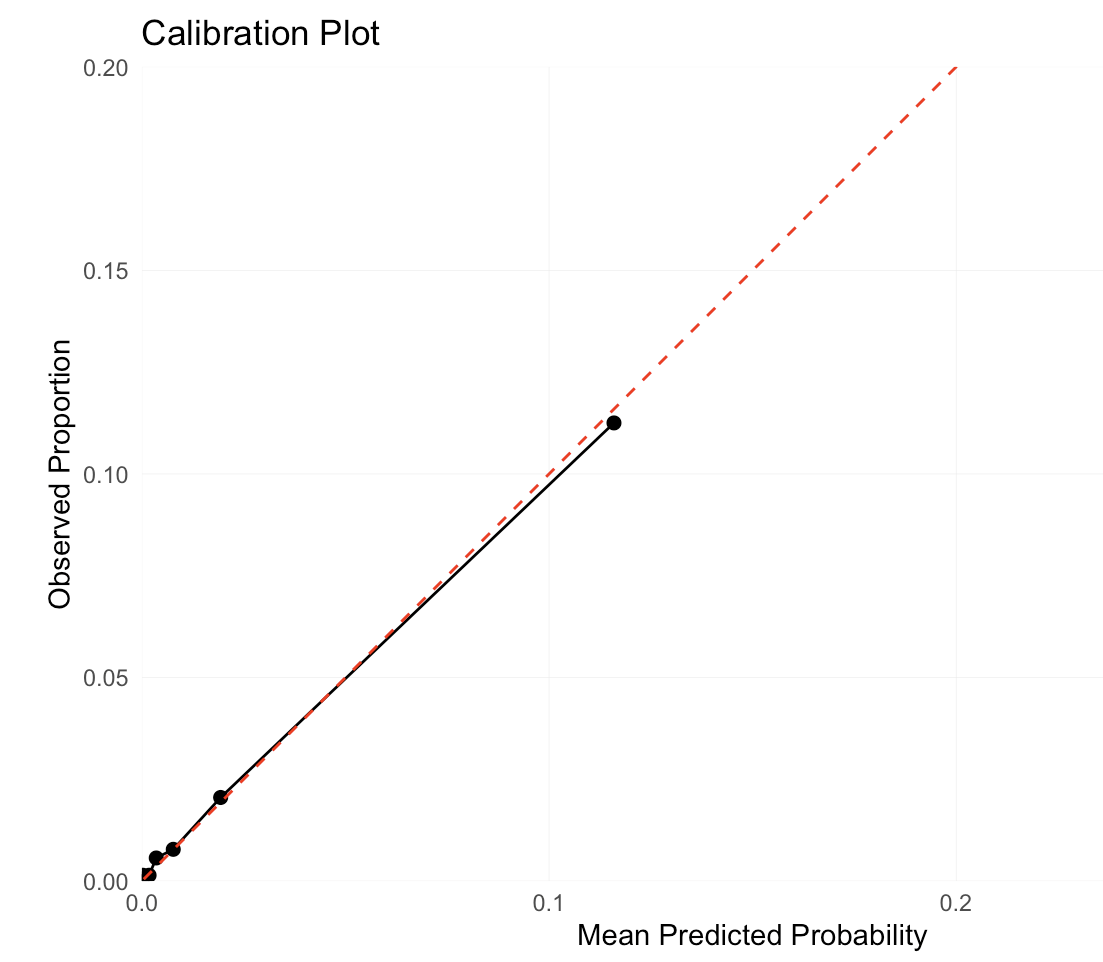

Supplement: Supplementary file 1 — Figure S1–S13 Calibration plots for the prediction of small‐for‐gestational age < 5th centile according to: maternal characteristics (Figure S1); estimated fetal weight centile (Figure S2); abdominal circumference centile (Figure S3); umbilical artery pulsatility index (Figure S4); middle cerebral artery pulsatility index (Figure S5); cerebroplacental ratio (Figure S6); uterine artery pulsatility index (Figure S7); estimated fetal weight centile and umbilical artery pulsatility index (Figure S8); estimated fetal weight centile and cerebroplacental ratio (Figure S9); estimated fetal weight centile, umbilical artery pulsatility index and middle cerebral artery pulsatility index (Figure S10); estimated fetal weight centile, umbilical artery pulsatility index and uterine artery pulsatility index (Figure S11); estimated fetal weight centile, uterine artery pulsatility index and cerebroplacental ratio (Figure S12); estimated fetal weight centile, middle cerebral artery pulsatility index and uterine artery pulsatility index (Figure S13). Figure S14–S26 Calibration plots for the prediction of fetal growth restriction according to: maternal characteristics (Figure S14); estimated fetal weight centile (Figure S15); abdominal circumference centile (Figure S16); umbilical artery pulsatility index (Figure S17); middle cerebral artery pulsatility index (Figure S18); cerebroplacental ratio (Figure S19); uterine artery pulsatility index (Figure S20); estimated fetal weight centile and umbilical artery pulsatility index (Figure S21); estimated fetal weight centile and cerebroplacental ratio (Figure S22); estimated fetal weight centile, umbilical artery pulsatility index and middle cerebral artery pulsatility index (Figure S23); estimated fetal weight centile, umbilical artery pulsatility index and uterine artery pulsatility index (Figure S24); estimated fetal weight centile, cerebroplacental ratio and uterine artery pulsatility index (Figure S25); estimated fetal weight centile, mid [file UOG-65-761-s001.docx]
